# Long-term outcomes after endoscopic treatment for Barrett’s neoplasia with radiofrequency ablation ± endoscopic resection: results from the national Dutch database in a 10-year period

**DOI:** 10.1136/gutjnl-2020-322615

**Published:** 2021-03-22

**Authors:** Sanne van Munster, Esther Nieuwenhuis, Bas L A M Weusten, Lorenza Alvarez Herrero, Auke Bogte, Alaa Alkhalaf, B E Schenk, Erik J Schoon, Wouter Curvers, Arjun D Koch, Steffi Elisabeth Maria van de Ven, Pieter Jan Floris de Jonge, Tjon J Tang, Wouter B Nagengast, Frans T M Peters, Jessie Westerhof, Martin H M G Houben, Jacques JGHM Bergman, Roos E Pouw, Dutch Barrett Expert Centers, A Karrenbeld

**Affiliations:** 1 Gastroenterology and Hepatology, Amsterdam UMC Locatie AMC, Amsterdam, North Holland, The Netherlands; 2 Gastroenterology and Hepatology, Sint Antonius Hospital, Nieuwegein, The Netherlands; 3 Gastroenterology and Hepatology, University Medical Center Utrecht, Utrecht, The Netherlands; 4 Gastroenterology and Hepatology, Isala Klinieken, Zwolle, Overijssel, The Netherlands; 5 Department of Gastroenterology and Hepatology, Catharina Hospital Eindhoven, Eindhoven, The Netherlands; 6 Gastroenterology and Hepatology, Erasmus University Medical Center, Rotterdam, The Netherlands; 7 Department of Gastroenterology and Hepatology, IJsselland Ziekenhuis, Capelle aan den IJssel, Zuid-Holland, The Netherlands; 8 Department of Gastroenterology and Hepatology, University of Groningen, University Medical Center Groningen, Groningen, The Netherlands; 9 Gastroenterology and Hepatology, Haga Hospital, Den Haag, Zuid-Holland, The Netherlands

**Keywords:** Barrett's oesophagus, Barrett's carcinoma, endoscopic procedures

## Abstract

**Objective:**

Radiofrequency ablation (RFA)±endoscopic resection (ER) is the preferred treatment for early neoplasia in Barrett’s oesophagus (BE). We aimed to report short-term and long-term outcomes for all 1384 patients treated in the Netherlands (NL) from 2008 to 2018, with uniform treatment and follow-up (FU) in a centralised setting.

**Design:**

Endoscopic therapy for early BE neoplasia in NL is centralised in nine expert centres with specifically trained endoscopists and pathologists that adhere to a joint protocol. Prospectively collected data are registered in a uniform database. Patients with low/high-grade dysplasia or low-risk cancer, were treated by ER of visible lesions followed by trimonthly RFA sessions of any residual BE until complete eradication of BE (CE-BE). Patients with ER alone were not included.

**Results:**

After ER (62% of cases; 43% low-risk cancers) and median 1 circumferential and 2 focal RFA (p25-p75 0–1; 1–2) per patient, CE-BE was achieved in 94% (1270/1348). Adverse events occurred in 21% (268/1386), most commonly oesophageal stenosis (15%), all were managed endoscopically. A total of 1154 patients with CE-BE were analysed for long-term outcomes. During median 43 months (22–69) and 4 endoscopies (1–5), 38 patients developed dysplastic recurrence (3%, annual recurrence risk 1%), all were detected as endoscopically visible abnormalities. Random biopsies from a normal appearing cardia showed intestinal metaplasia (IM) in 14% and neoplasia in 0%. A finding of IM in the cardia was reproduced during further FU in only 33%, none progressed to neoplasia. Frequent FU visits in the first year of FU were not associated with recurrence risk.

**Conclusion:**

In a setting of centralised care, RFA±ER is effective for eradication of Barrett’s related neoplasia and has remarkably low rates of dysplastic recurrence. Our data support more lenient FU intervals, with emphasis on careful endoscopic inspection. Random biopsies from neosquamous epithelium and cardia are of questionable value.

**Netherlands trial register number:**

NL7039.

**Significance of this study**

**What is already known on this subject?**

- Endoscopic treatment by means of endoscopic resection (ER) and radiofrequency ablation (RFA) is safe and effective for Barrett’s oesophagus-related neoplasia.

**What are the new findings?**

- After successful RFA treatment, the long-term risk for dysplastic recurrence is remarkably low: 1% per person year. All recurrences are detected as endoscopic abnormalities and not through random biopsies. There was no association between 3-monthly endoscopies in year 1 and improved patient outcomes. We also found no association between intestinal metaplasia in a normal appearing cardia and future dysplastic recurrence.

**How might it impact on clinical practice in the foreseeable future?**

- We suggest two important recommendations for post-treatment surveillance: (1) there is no need for frequent 3-monthly endoscopies during the first year after treatment; and (2) careful inspection is the most important aspect of follow-up endoscopies and random biopsies may be abandoned.

## Introduction

Endoscopic eradication treatment (EET) is an established treatment approach for eradicating Barrett’s oesophagus (BE) with early neoplasia. EET is generally a multimodal treatment consisting of endoscopic resection (ER) in case of visible lesions, followed by eradication of the residual flat BE segment, to minimise the risk of metachronous dysplasia. For the latter, radiofrequency ablation (RFA) is the most commonly used technique. Current clinical guidelines unanimously recommend this effective and safe two-step approach as standard of care.^1–3^

Landmark studies consistently report excellent efficacy, with complete eradication of all neoplasia as well as complete eradication of all BE in 74%–98% of patients.^4–7^ However, the long-term durability remains poorly characterised. Some studies have reported long-term outcomes, but were limited by small sample size, heterogeneous treatment and follow-up (FU) protocols, and/or different definitions for recurrence. Consequently, reported rates for dysplastic recurrence vary widely from 1% to 20% per person year.^4 5 8–13^

EET for BE related dysplasia and early cancer in the Netherlands is uniquely organised, with centralisation of care in Barrett Expert Centers (BECs). All patients are referred to a BEC, where care is provided by experienced endoscopists and pathologists, all of whom participated in joint training programmes. All centres adhere to a joint treatment and FU protocol and difficult cases are discussed in regular interdisciplinary meetings. Data on treatment and outcomes of all patients treated in the BECs are registered in a uniform database. A joint research network has been established for studies in the field of pathology,^14–17^ imaging,^18–20^ and treatment^4 5 9 21–26^ for early BE neoplasia. The aim of the current study was to report the short-term and long-term outcomes for all patients treated for BE with dysplasia and/or early cancer in the Netherlands, according to a uniform EET protocol including RFA.

## Methods

The BEC registry is an ongoing, multicentre initiative designed to establish outcomes of patients undergoing EET for early BE neoplasia in a setting of centralised care (Netherlands Trial Register, NL7039, online supplemental table S1). The registry includes data for all patients who underwent endoscopic treatment for early BE neoplasia in the Netherlands since 2008, when RFA was introduced into regular clinical practice. The Dutch patient federation for cancer of the digestive tract (‘Stichting voor patiënten met kanker aan het spijsverteringskanaal’) was involved in the design, reporting and dissemination plans of our study.

10.1136/gutjnl-2020-322615.supp1 Supplementary data

### Study population

**Figure 1:**
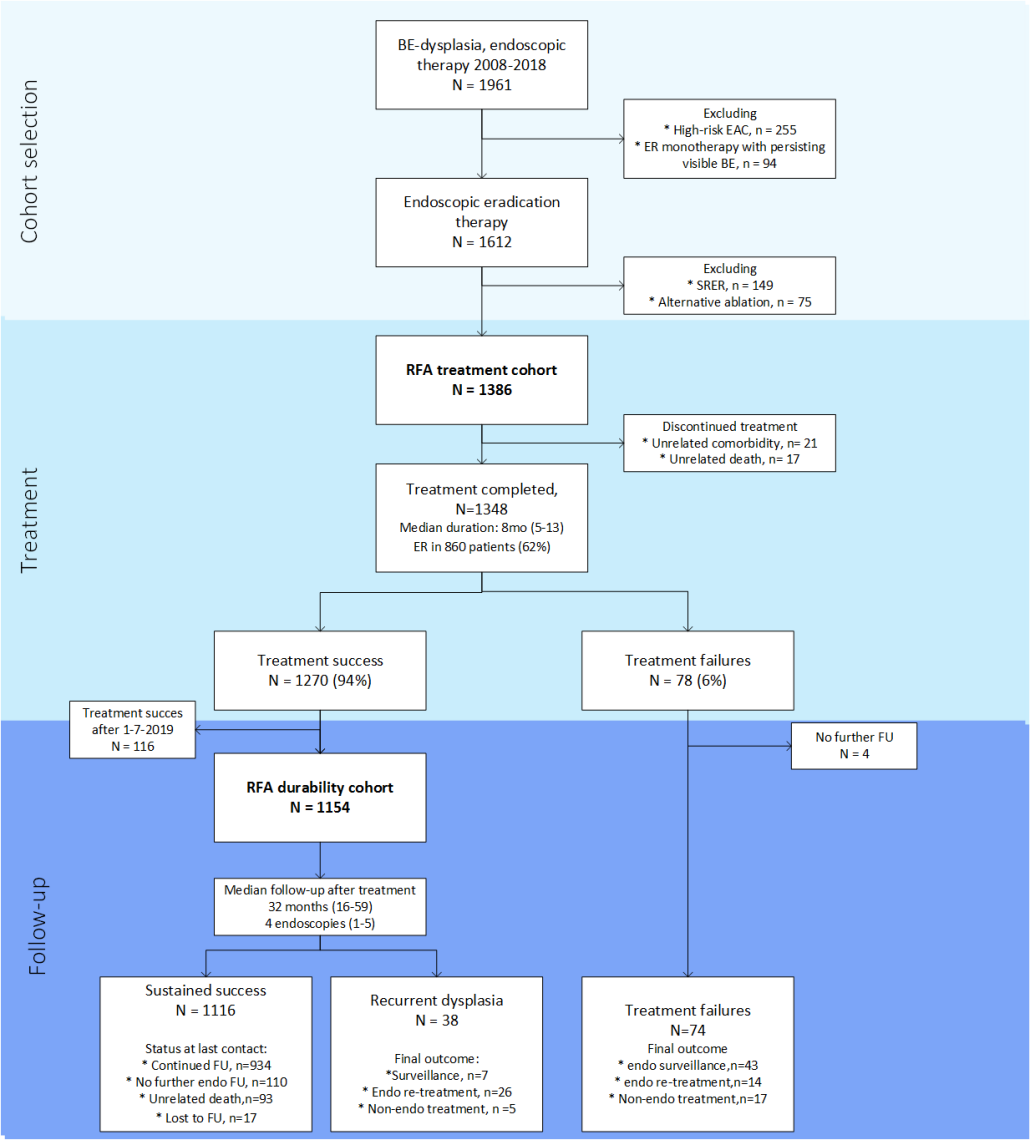
Patient flow. BE, Barrett’s oesophagus; EAC, oesophageal adenocarcinoma; ER, endoscopic resection; FU, follow-up; RFA, radiofrequency ablation; SRER, stepwise radical endoscopic resection.

For the current study, all patients with BE and confirmed low-grade dysplasia (LGD), high-grade dysplasia (HGD) or low-risk oesophageal adenocarcinoma (LR EAC) (mucosal or superficial submucosal (sm1) cancer, well to moderately differentiated, without lymphovascular invasion, no tumour invasion (R0) in the vertical resection margin), who underwent at least 1 RFA treatment between 1 January 2008 and 31 December 2018, were included in the *‘RFA treatment cohort*’ (figure 1). Non-dysplastic Barrett’s oesophagus (NDBE) is not an accepted indication for RFA in our country and these patients were not included in our cohort.

**Figure 2:**
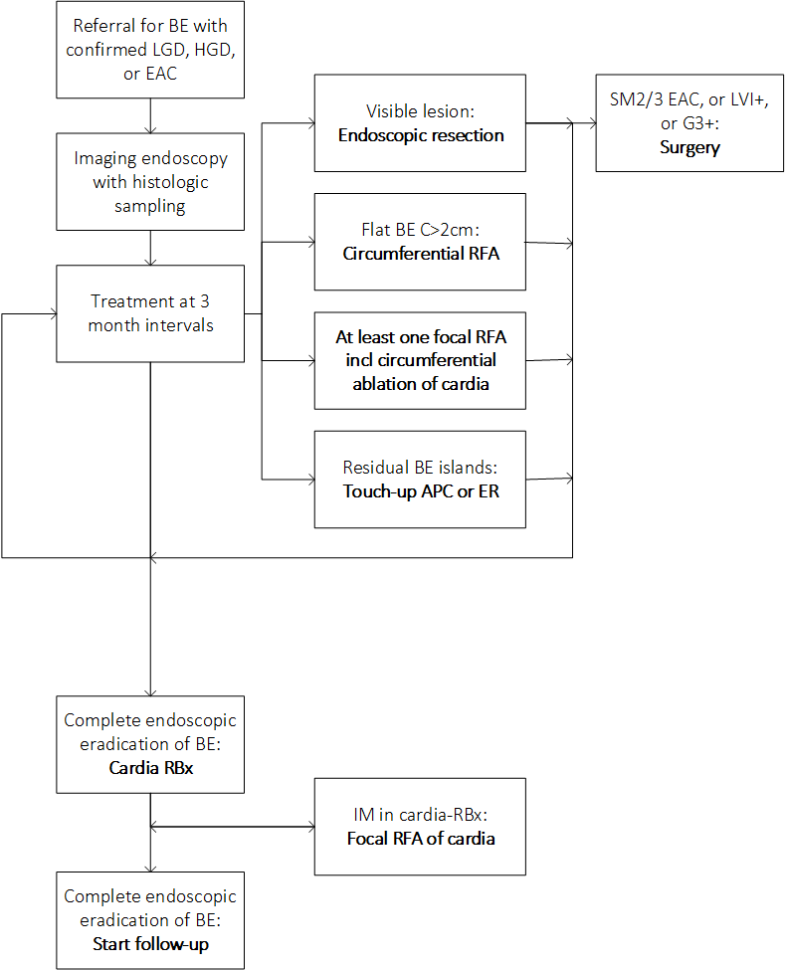
Treatment protocol. Treatment protocol followed by all Barrett Expert Centers in the Netherlands. APC, argon plasma coagulation; BE, Barrett’s oesophagus; ER, endoscopic resection; EAC, esophageal adenocarcinoma; HGD, high-grade dysplasia; LGD, low-grade dysplasia; IM, intestinal metaplasia; RFA, radiofrequency ablation.

The *‘RFA durability cohort*’ (figure 1) was defined as all patients with successful EET, defined as complete endoscopic eradication of BE (CE-BE), with at least 1-year FU at the moment of data collection.

We excluded 255 cases (figure 1) with high-risk EAC in their ER specimen (ie, deep submucosal invasion (sm2-3), poor differentiation, lymphovascular invasion or invasion of cancer (R1) in the vertical resection margin).

We also excluded cases (n=94; figure 1) in whom—after the ER—no further attempts were at CE-BE for various reasons, mainly limited life expectancy.^27^ Finally, we excluded 224 patients (figure 1) in whom other techniques than RFA were used to achieve CE-BE, either stepwise radical endoscopic resection (SRER; n=149), hybrid-APC (n=43), endorotor (n=20), cryoballoon ablation (n=9) or other techniques (n=3).

Part of the patients included in the current study have been treated in context of a prospective clinical trial and were therefore mentioned in prior published work.^4 5 9 24 28 29^

### Treatment protocol

Patients who were referred with histologically confirmed LGD, HGD or LR-EAC underwent a dedicated imaging endoscopy using high-definition endoscopy. The oesophagus was carefully inspected with documentation of the Prague C&M criteria,^30^ presence of visible lesions or other abnormalities such as oesophagitis or stenosis. Visible lesions were removed with ER, per default using cap-based ER and endoscopic submucosal dissection was used for special indications, that is, bulky lesions that could not be sucked in the ER cap, or lesions with a suspicion of submucosal invasion. Patients with limited life expectancy underwent ER monotherapy and no RFA, with surveillance of the remaining BE segment. For all other patients, RFA (Barrx system, Medtronic, Minneapolis, Minnesota, USA) was performed at 3–4 month intervals to eradicate flat BE as described previously^4^ (figure 2). Touch-up treatment for any residual flat, non-neoplastic BE areas that persisted after RFA treatment (including at least 1 focal RFA of the GE-junction) was allowed, using ER (for areas>5 mm) or a maximum of two APC sessions for areas<5 mm. If biopsies from the GE-junction showed persisting IM after RFA, one additional focal RFA of the GE junction was allowed. Residual BE could also be kept under endoscopic surveillance, at the discretion of the endoscopist.

When complete endoscopic eradication of all visible BE was achieved, the oesophagus was sampled to confirm eradication of IM. Initially, biopsies were obtained from neosquamous epithelium (NSE) over the length of the initial BE segment (4 quadrants every 2 cm) and from the cardia (ie, <5 mm distally from the neosquamocolumnar junction). From 2013, the NSE biopsies were abandoned due to low diagnostic yield and emerging evidence that adequate endoscopic inspection provided more clinically relevant information than random NSE biopsies.^31^

### Follow-up protocol

Endoscopic FU consisted of high-definition endoscopy with optical chromoendoscopy, with changing intervals and sampling methods over time. Initially, FU endoscopies were performed every 3 months in the first year, followed by annual endoscopies in years 2–5, and then one endoscopy in every 2–3 years. However, from 2015, we abandoned the quarterly endoscopies in the first year, due to low yield of clinically relevant findings.

From 2008 till 2013, 4 quadrant random biopsies were obtained from the entire NSE at 2 cm intervals and from the cardia during every FU endoscopy. In 2013, we abandoned the NSE biopsies and in 2016 we abandoned the random biopsies from the cardia, due to low yield of clinically relevant findings. Residual BE including an irregular Z-line, visible lesions, or other abnormalities always remained an indication for histological sampling.

Treatment for recurrent non-dysplastic BE was per endoscopist’s discretion and based on the estimated risk for progression, and a patient’s age and comorbidity. Recurrent (or persisting) BE islands were treated with re-APC.

During treatment and FU, all patients were prescribed high-dose proton-pump inhibitor therapy twice daily, supplemented with sucralfate suspension after every meal for 2 weeks after each therapeutic endoscopy.

### Histological analysis

Histological evaluation of all ER specimens and biopsies obtained at baseline, during treatment and at least the first FU endoscopy was performed by a dedicated BE expert pathologist. The training of the BEC pathologists has been described in detail elsewhere.^15 17 32^

### Endpoints RFA treatment cohort

Primary effectiveness endpoint:

1. Proportion of patients with CE-BE at the first endoscopy after the treatment phase. A patient was considered a failure for this endpoint if residual endoscopically visible BE persisted after completing the treatment protocol including touch-up treatment, and/or if dysplasia persisted, including dysplasia in cardia biopsies without visible BE. IM in cardia biopsies in the absence of endoscopically visible BE was not considered a treatment failure.^4^ All patients in the treatment cohort who completed the treatment protocol were included for this analysis (‘per protocol population’). We divided treatment failures into two groups: (a) real treatment failures in whom>20% of the initial BE persisted and/or in whom neoplasia persisted; and (b) patients with >80% of the initial BE removed and complete eradication of neoplasia, in whom an elective decision was made to withhold further treatment.

Secondary effectiveness endpoints:

1. Differences in outcomes over time.
2. Progression to advanced EAC that exceeded boundaries for curative endoscopic treatment.
3. Complications (oesophageal stenosis, bleeding, perforation, death).

### Endpoints RFA durability cohort

Primary durability endpoint:

1. Proportion of patients with sustained eradication of LGD, HGD and EAC during long-term endoscopic FU. A patient was considered a failure for this endpoint if recurrent LGD, HGD or EAC was detected in the oesophagus or cardia, or if lymph node or distant metastasis from EAC were found during FU. Failure for this endpoint was categorised into three groups according to the severity of recurrent disease: (a) LGD in a normal appearing cardia without recurrent BE; (b) recurrent BE with LGD/HGD/EAC amendable for curative endoscopic treatment; and (c) advanced EAC that exceeded boundaries for curative endoscopic treatment.

Secondary durability endpoints:

1. Sustained eradication of HGD and EAC (recurrent LGD was considered as success).
2. Progression to advanced EAC that exceeded boundaries for curative endoscopic treatment.
3. Recurrence of non-dysplastic BE.
4. Diagnostic yield for FU endoscopies and random biopsies.
5. Association between frequent endoscopies in the first FU year and recurrence.
6. Association between IM in the cardia and recurrence.
7. Unrelated mortality rates and causes of death.

Detailed definitions for our endpoints are provided in online supplemental table S2.

### Data collection

The BEC registry, a joint database that includes all treated patients in the Netherlands, was initiated in 2016. Patients were identified from the prospective annual registrations of treatment outcomes, prospective databases and/or patient lists at each centre. All relevant data regarding baseline characteristics, imaging, treatment and FU were retrospectively recorded from the endoscopy and pathology reports in the electronic patient files that were kept in each individual centre, and which were standardised from the beginning of the joint treatment protocol in 2008. All data were coded and merged in a joint, online database (Castor EDC), with a separately kept patient identification file.

The BEC registry was merged with the non-public microdata from Statistics Netherlands for survival outcomes, including date and cause of death.

### Data management

Medical students in the final year of their degree reviewed all endoscopy and pathology reports for data collection under frequent supervision and after standardised training in the subject and the database. A second, independent assessment by a dedicated research fellow (MD) was done for a random 50% of the patient population. Additionally, all patients meeting primary or secondary endpoints had source data verified by a research fellow (MD) and were discussed during meetings with the study team (SvM, EN, RP, JB). All fields were examined for missing data, strange values or outliers, and these were completed or corrected where possible. All authors had access to the study data and reviewed and approved the final manuscript.

### Statistics

Results of the descriptive analyses are presented as counts and proportions for categorical variables and median and ranges between the 25th and 75th percentile, or mean and SD for continues variables with skewed or normal distribution, respectively. CIs (2.5th; 97.5th percentile) were obtained using bootstrapping. Categorical variables were compared using a X^2^ test or Fisher’s exact test, and continuous variables with a Student’s t-test or Mann-Whitney U test.

The durability of eradication of dysplasia was estimated with the use of the Kaplan-Meier method. The HR for recurrent dysplasia was estimated with the use of a Cox proportional hazards model. Data for patients were censored at the last endoscopic FU. To assess causal associations, cox proportional hazard models were adjusted for age, gender, length of BE, worst histology at baseline, presence of a reflux stenosis, and presence of incident lesions during RFA treatment.

Median overall survival was estimated with use of the Kaplan-Meier. Patients were censored at the date patient was last known to be alive. Recurrence and survival were combined with the use of a cumulative incidence curve. Statistical analysis were performed using Rstudio for windows (V.3.6.1).

## Results

### Baseline characteristics

A total of 1386 patients underwent at least one RFA treatment between 2008 and 2018 and were analysed for safety and effectiveness (*‘RFA treatment cohort”*) (figure 1). Patient characteristics are shown in table 1.

**Table 1:** Baseline characteristics

	RFA treatment cohort N=1386	RFA durability cohort N=1154
**Demographics**		
Male gender, n (%)	1122 (81)	947 (82)
Age, years, mean (±SD)	65 (10)	64 (9)
BMI, kg/m^2^, mean (±SD)	28 (4)	28 (4)
**BE history**		
Prior fundoplication, n (%)	23 (2)	21 (2)
Surveillance history, n (%) In years, median (P25–P75)	892 (64) 4 (2–8)	759 (66) 3 (0–8)
**Imaging**		
Hiatal hernia, n (%) In cm, mean (±SD)	1321 (95) 3 (2–4)	1099 (95) 3 (2–4)
Oesophagitis, n (%)	49 (4)	38 (3)
Stenosis, n (%)	49 (4)	42 (4)
Circumferential BE, median (P25–P75)	2 (1–6)	2 (0–5)
Maximum BE, median (P25–P75)	5 (3–8)	4 (3–7)
Visible lesion, n (%)	860 (62)	718 (62)
Primary Paris type, n (%)		
0-Ip/s	81 (11)	63 (9)
0-IIa	490 (69)	419 (58)
0-IIb	111 (16)	90 (13)
0-IIc	29 (4)	22 (3)
	149 missing	124 missing
Size, mm, median (P25–P75)	15 (10–20)	15 (10–20)
**Pathology**		
Worst overall histology, n (%)		
LGD	375 (27)	306 (27)
HGD	422 (30)	362 (31)
LR-EAC	589 (43)	486 (42)
**Treatment**		
Endoscopic resection, n (%)	860 (62)	718 (62)
Cap-based ER, n (%)	839 (61)	688 (60)
ESD, n (%)	31 (2)	20 (2)
RFA treatment		
C-RFA, median (P25–P75)	1 (0–1)	1 (0–1)
F-RFA, median (P25–P75)	2 (1–2)	2 (1–2)
Total RFA, median (P25–P75)	2 (1–3)	2 (1–3)
Patients with >2 C-RFA, n (%)	9 (0.6)	6 (0.5)
Patients with >4 total RFA, n (%)	57 (4)	44 (4)
Touch-up APC, n (%)	519 (37)	462 (40)
Touch-up ER, n (%)	80 (6)	74 (6)
ER for incident lesion, n (%)	69 (5)	44 (4)

APC, argon plasma coagulation; BE, Barrett’s oesophagus; BMI, body mass index; C-RFA, circumferential RFA; EAC, esophageal adenocarcinoma; EMR, endoscopic mucosal resection; ER, endoscopic resection; ESD, endoscopic submucosal dissection; F-RFA, focal RFA; GEJ, gastroesophageal junction; HGD, high-grade dysplasia; LGD, low-grade dysplasia; LR-EAC, Low-risk esophageal adenocarcinoma.

### Treatment cohort outcomes

CE-BE (ie, a complete endoscopic eradication of all visible BE) at the end of the treatment phase was achieved in 94% of patients who completed the treatment protocol (95% CI 93 to 95) (1270/1348). This proportion was constant over time (online supplemental figure S1). Of the 1270 patients with CE-BE, 85 (7%) had persisting IM in biopsies obtained from a normal appearing cardia.

10.1136/gutjnl-2020-322615.supp2 Supplementary data

In 62% of patients, a visible lesion was removed with ER before RFA. This proportion differed along with the worst histological diagnosis at baseline: 17% of patients with LGD underwent baseline ER (62/375), compared with 53% of HGD patients (225/422) and 99% of patients with EAC (583/589). Six patients had EAC in a random biopsy, without visible lesions, and underwent RFA monotherapy, all were treated before 2015. For HGD patients, this proportion showed a significant increase over the years (47% before 2013 and 59% thereafter, p0.01; online supplemental figure S1).

A total of 68 (5%) patients were noted to have a neoplastic lesion after RFA was started (‘incident lesion’) that was removed with ER and showed HGD (n=26) or EAC (n=42). Baseline histology for these patients was HGD (n=27), or EAC (n=41). The incident lesion harboured a worse diagnosis than at baseline in 20 patients (20/1386; 1%).

Treatment consisted of median 1 circumferential (p25–p75 0–1) and two focal (p25–p75 1–2) RFA treatments per patient and was followed by touch-up ER (80/1386; 6%) and/or APC (519/1386; 37%) for residual BE areas.

#### Treatment failures

Seventy-eight patients (78/1348; 6%) had remaining Barrett’s mucosa and/or dysplasia, and were defined as failures after median 10 (p25–p75 5–22) months of treatment. In 34 failures (34/78; 44%), over 80% of the initial BE had been removed, and an elective decision was made to withhold further treatment. These patients had median C1M2 residual BE with non-dysplastic IM (n=21) or LGD (n=13) (online supplemental table S3). During mean surveillance of 49 months with 3 endoscopies per patient after treatment was stopped, 6 (18%) developed a visible lesion. All were detected at early stages and were curatively treated endoscopically with ER for HGD (n=4) or mucosal EAC (n=2).

The other 44 failures (44/78; 56%) were real treatment failures in whom CE-BE could not be achieved, due to poor squamous regeneration (n=27) or progression to disease that exceeded boundaries for curative endoscopic treatment (n=17). These patients had median C5M7 residual BE with non-dysplastic IM (n=16), LGD (n=8), HGD (n=11), or EAC (n=9) (online supplemental table S3). The 17 patients (17/1386; 1%) who progressed to disease that exceeded the curative indication of endoscopic treatment developed high-risk EAC (n=7, all diagnosed after ER for an incident lesion) or new visible abnormalities that could not be removed with ER due to multifocality (n=9) or a persisting visible lesion that could not be removed with ER due to post-treatment fibrosis (n=1). Nine patients underwent esophagectomy and remained free of disease up to the moment of data collection (n=7) or died due to unrelated causes (n=2). The other eight patients were unfit for major surgery and had EAC-related death (n=4); unrelated death (n=2); or were alive at the moment of data collection (n=2). Twelve of these 17 cases were identified at baseline as complicated cases due to BE segment >10 cm, severe reflux oesophagitis, and/or multifocal neoplasia (online supplemental table S4).

The majority of the real treatment failures was identified early in the treatment phase. The median time between first treatment and decision to stop further treatment was 8 months. In two-thirds (29/44, 67%), treatment was stopped within the first 12 months, in 10 (23%) between 12 and 18 months, and in 4 (9%) after 18 months.

#### Complications

Oesophageal stenosis requiring endoscopic dilatation was the most common complication and occurred in 15% of patients (95% CI 13 to 17) (210/1386) (table 2). In 170 cases (170/1386; 12%), stenosis was resolved after 5 or less dilatations (median 2), but 40 patients (40/1386; 3%) developed a severe stenosis that required median 9 endoscopic dilatations. Additional incision therapy was required in 10 patients and esophageal stent placement in 4. All stenosis were managed endoscopically. Most severe stenosis occurred after extensive ER followed by RFA, but 12 patients (12/1386; 0.9%) developed a severe stenosis after a single circumferential RFA. Increasing BE length, prior ER and more extensive prior ER were risk factors for stenosis (online supplemental table S5).

**Table 2:** Safety outcomes

	Total patients N=1386
At least 1 complication, n (% (95% CI))	268 (21 (19 to 23))
**Stenosis**	
Incidence, n (% (95% CI))	210 (15 (13 to 17))
Severity*, n (% (95% CI))	
Mild/moderate	170 (12 (11 to 14))
Severe	40 (3 (2 to 4))
**Post-procedural bleed**	
Incidence, n (% (95% CI))	52 (4 (3 to 5))
Severity*, n (% (95% CI))	
Mild	19 (1 (1 to 2))
Moderate	25 (2 (1 to 3))
Severe	8 (0.5 (0.3 to 1))
Cause, n	
ER	29
RFA	23
**Perforation**	
Incidence, n (% (95% CI))	11 (0.8 (0.4 to 1))
Severity*, n (% (95% CI))	
Mild	5 (0.4 (0.1 to 0.9))
Moderate	6 (0.4 (0.2 to 1)
Severe	–
Cause, n	
ER	6
Endoscopic dilatation	5

*Adverse events were graded as ‘mild’ (unplanned hospital admission, hospitalisation <3 days, haemoglobin drop <3 g, no transfusion), ‘moderate’ (4–10 days hospitalisation,<4 units blood transfusion, repeat endoscopic intervention, radiological intervention), ‘severe’ (hospitalisation >10 days, intensive care unit (ICU) admission, need for surgery, >4 units blood transfusion, in the case of stenosis: >5 dilatations, stent placement or incision therapy) or ‘fatal’ (death attributable to procedure <30 days or longer with continuous hospitalisation). See online supplemental table S2 for more definitions.

ER, Endoscopic resection; RFA, Radiofrequency ablation.

The bleeding rate for RFA was 2% per procedure (95% CI 1 to 2) or 4% per patient (95% CI 3 to 5). No perforations occurred after RFA. Perforations occurred in 11 patients (1% (95% CI 0 to 1)) after ER (n=6) or endoscopic dilatation for oesophageal stenosis (n=5), all were managed conservatively or with endoscopic intervention.

There were no procedure related deaths.

### Durability cohort outcomes

One thousand one hundred fifty-four patients who had a complete eradication of BE (CE-BE) on RFA, were analysed for long-term outcomes. The median duration of endoscopic FU (ie, until the last FU endoscopy) was 43 (p25–p75 22–69, minimum 8) months after baseline and 32 (16–59) months after the last treatment (total time at risk 3706 person years) with median 4 (1–5) FU endoscopies per patient. A substantial number of patients had long-term FU: 317 patients had FU ≥5 years and 148 patients had FU ≥7 years after achieving CE-BE. The majority of patients was still under endoscopic surveillance at the moment of data collection (n=934). In 203 patients, endoscopic FU was stopped due to age, comorbidity or death, median 37 months after the last treatment. Seventeen patients (1%) were lost to FU after mean 34 months of endoscopic surveillance with median 3 FU endoscopies.

**Figure 3:**
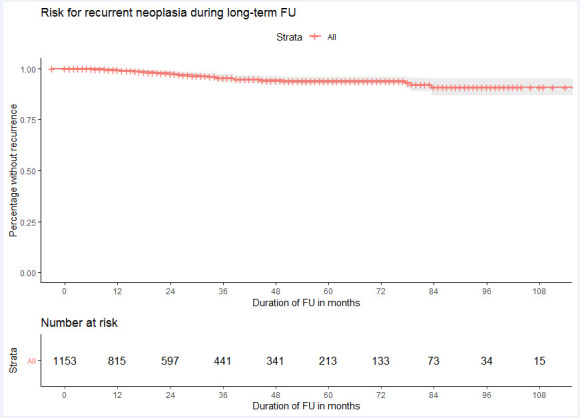
Long-term outcomes. Kaplan-Meier curve for the risk for recurrent dysplasia during follow-up (FU) based on the ‘RFA durability cohort’. A patient was considered a failure for the endpoint if recurrent dysplasia was found, irrespective of whether curative endoscopic retreatment was performed. Patients were censored at the last FU endoscopy at the moment of data collection.

During FU, recurrence of LGD, HGD or EAC occurred in 38 (38/1154; 3%) patients (annual risk 1.0% (95% CI 0.8 to 1.4)) (figure 3). A total of 24 patients had recurrent HGD/EAC (24/1154; 2%; annual risk 0.7 (95% CI 0.4 to 1.0)).

Recurrences were categorised in three grades: (1) LGD in a normal appearing cardia (9/38; 24%); (2) recurrent BE with dysplasia/EAC (24/38; 63%); and (3) advanced EAC that exceeded boundaries for curative endoscopic treatment (5/38; 13%) (table 3, online supplemental figure S2). Patients in category 1 underwent treatment (n=1) or surveillance without progression (n=8). All patients in category 2 underwent successful endoscopic treatment and CE-BE was re-established in all. Of the five patients with progression to advanced EAC (5/1154, 0.4%, annual risk 0.1% (95% CI 0.1 to 0.3)), four patients underwent surgery (T1bN1; T1bN1, T1N2, T1bN0), of which three patients later died from metastasised disease. A single patient had metastasised disease without intraluminal recurrence at the moment recurrence was diagnosed. Three of the five patients were identified at baseline as complicated cases with BE segment >10 cm, severe reflux oesophagitis and/or multifocal neoplasia (online supplemental table S4). In total, 8 patients (8/1154; 0.7%) had a worse disease stage during FU than at baseline staging.

**Table 3:** Recurrences

	LGD at GEJ N=9	Recurrent dysplasia/EAC N=24	Advanced EAC N=5
**Initial BE**			
Length, median (P25–P75)	C6M7 (4–9; 5–9)	C3M5 (1–7; 3–9)	C8M10 (5–11; 7–12)
Histology, n (%)			
LGD	1 (11)	3 (13)	–
HGD	6 (67)	5 (21)	2 (40)
LR-EAC	2 (22)	16 (67)	3 (60)
Severe reflux, n (%)	3 (33)	1 (4)	3 (60)
**Treatment**			
Baseline ER, n (%)	5 (56)	19 (79)	4 (80)
N C-RFA, median (P25–P75)	1 (1–2)	1 (0–1)	1 (1–2)
N F-RFA, median (P25–P75)	2 (2–3)	2 (1–2)	2 (1–3)
**FU**			
Prior IM in cardia, n (%)	2 (22)	1 (4)	0
N FU endoscopies before recurrence, median (P25–P75)	3 (1–5)	4 (2–5)	2 (2–3)
Months between last treatment and recurrence, median (P25–P75)	31 (17–45)	31 (23–47)	25 (18–39)
Months between last FU endsoscopy and recurrence, median (P25–P75)	11 (9–13)	12 (10–15)	12 (7–17)
**Recurrence**			
Location, n (%)			
Cardia	9 (100)	4 (17)	1 (25)*
Tubular	–	20 (83)	3 (75)
Detection	Cardia RBx	Visible BE a/o lesion	Visible BE a/o lesion*
Histology, n (%)			
LGD	9 (100)	5 (21)	
HGD		7 (29)†	
LR-EAC		12 (50)†	
HR-EAC			5 (100)†

*A single patient developed symptomatic, metastasized disease without abnormalities in the oesophagus.

†A worst histological grade during FU as compared with baseline, was found in eight patients in total. Three patients with baseline LGD who developed HGD (n=1) or LR-EAC (n=2) and in all five patients who developed HR-EAC during FU.

BE, Barrett’s oesophagus; C-RFA, circumferential RFA; EAC, oesophageal adenocarcinoma; ER, endoscopic resection; F-RFA, focal RFA; FU, follow-up; HGD, high-grade dysplasia; IM, intestinal metaplasia; LGD, low-grade dysplasia; RBx, Random biopsies.

Recurrence occurred median 31 months^19–43^ after CE-BE (figure 3). The majority of recurrences developed in the tubular oesophagus (24/38; 62%); either in short segment BE (median C1M2) or in small BE islands, always within the extent of the initial BE segment (online supplemental figure S3). The remaining 38% (14/38) occurred at the cardia. All recurrences in categories 2 and 3 were detected as endoscopic abnormalities (recurrent BE and/or visible lesion). No recurrent HGD/EAC was diagnosed solely on random biopsies.

10.1136/gutjnl-2020-322615.supp4 Supplementary data

#### Recurrent non-dysplastic BE

During FU, recurrent NDBE occurred in 109 patients (9% (8–11)), the majority of which had diminutive BE islands (84/1154; 7%). Twenty-seven patients (27/1154; 2%) developed recurrent BE tongues of limited size (median C0M2), in all cases of lesser extent than the initial BE (online supplemental table S6). One patient (1/109; 1%) progressed to LGD in a recurrent C1M2 BE. No patient with recurrent BE progressed to HGD/EAC during a median FU of 24 months with median 2 endoscopies.

Recurrent BE tongues were detected after median 38 months, whereas BE islands were detected significantly shortly after treatment (median 15 months, P0.02). The annual risk for BE islands was 3% (2–4) in years 1–2 and 1% (1–1) in the years thereafter. The risk for recurrent BE tongues was 0.4% (95% CI 0.2% to 0.8%) in the first 2 years and 1% (95% CI 1% to 2%) in the years thereafter (online supplemental figure S4 and S5).

10.1136/gutjnl-2020-322615.supp5 Supplementary data

10.1136/gutjnl-2020-322615.supp6 Supplementary data

#### Diagnostic yield of FU endoscopies

Overall, a total of 3889 FU endoscopies was performed in 1154 patients. The diagnostic yield for detection of recurrent LGD/HGD/EAC was 1.0% (95% CI 0.7% to 1.3%) (38/3889) per endoscopy and 0.6% (95% CI 0.4 to 0.9) for recurrent HGD/EAC (24/3889).

Patients in whom CE-BE was achieved before 2015 (n=393) underwent 3-monthly endoscopies in the first year of FU (ie, FU at 0–3–6–9–12 months from CE-BE), whereas the remaining 761 patients had the first FU endoscopy performed after 1 year (ie, at 0–12 months from CE-BE). In multivariate cox analysis, no significant association was found between the frequency of FU in year 1 and dysplastic recurrence during the first 30 months (adjusted HR 1.6 (95% CI 0.6 to 4.1)). During long-term FU, no significant association was found between the frequency of FU in year 1 and progression to advanced neoplasia (adjusted HR 0.8 (95% CI 0.1 to 5.8)) (online supplemental table S7).

#### Random sampling from neosquamous epithelium

A total of 8588 random biopsies were obtained from the NSE in 376 patients during 924 FU endoscopies. Buried BE was found in 10 biopsies; in 1% of all endoscopies (95% CI 1 to 2) and 0.1% of all biopsies (95% CI 0.1 to 0.2) (table 4). None of the buried BE samples showed signs of dysplasia and during a median FU of 4 years and a median of 4 endoscopies after buried BE was noted, the finding was not reproduced and none of the patients showed dysplastic progression.

**Table 4:** Diagnostic yield and relevance of random biopsies during follow-up (FU)

Finding	Cumulative incidence			Relevance				
	Patient rate % (95% CI) n/N*	Endoscopy rate % (95% CI) n/N†	Biopsy rate % (95% CI) n/N	FU‡; Years Median (P25–P75)	FU‡, N endoscopies Median (P25–P75)	Reproduced % (95% CI) n/N	Progression to LGD/HGD/EAC % (95% CI) n/N	Progression to HGD/EAC % (95% CI) n/N
NSE random biopsies								
Buried IM	2.7 (1.5 to 4.8) (10/376)	1.1 (0.6 to 2.0) (10/924)	0.1 (0.1 to 0.2) (10/8588)	4 (4 to 5)	4 (4 to 5)	0 (0 to 3.4) (0/10)	0 (0 to 3.4) (0/10)	0 (0 to 3.4) (0/10)
Cardia random biopsies								
IM	13.8 (11.5 to 15.5) (150/1121)	7.2 (6.3 to 8.3) (198/2733)	NA	3 (2 to 4)	3 (2 to 4)	33.3 (25.8 to 41.8) (43/129)§	2.3 (0.1 to 7.2) (3/129)	0 (0 to 2.9) (0/129)
LGD	0.81 (0.42 to 1.5) (9/11 121)	0.73 (0.46 to 1.15) (20/2733)	NA	2 (2 to 5)	2 (2 to 4)	75.0 (35.6 to 95.6) (6/8)¶	NA	0 (0 to 40.2) (0/8)

The diagnostic yield of random biopsies from NSE and cardia and long-term follow-up of abnormal findings.

*N = patients with at least 1 endoscopy with sampling from NSE or cardia.

†N = Endoscopies with sampling from NSE or cardia.

‡Median FU after detection of buried BE; IM; of LGD.

§N=patients with IM in the cardia; either at end of treatment (n=78) or during FU (n=72). Patients with treatment (n=9) or no FU (n=12) were excluded.

¶A single patient underwent additional RFA and was not included for the FU analysis.

**Adjusted for potential confounders age, gender, length of BE, worst pathology at baseline, reflux stenosis, incident lesion.

BE, Barrett’s oesophagus; IM, intestinal metaplasia; LGD, low-grade dysplasia; NSE, neosquamous epithelium; RBx, random biopsies; RFA, radiofrequency ablation.

In 2013, we stopped obtaining random NSE biopsies. Outcomes before 2013 (annual recurrence risk 1.3 (95% CI 0.5 to 2.1)) did not differ significantly from those after 2013 (annual recurrence risk 1.0 (95% CI 0.6 to 1.3)) (p 0.56).

#### Random sampling from the cardia

Random biopsies from a normal appearing cardia were obtained during 2733 FU endoscopies in 1121 patients (table 4). Non-dysplastic IM was found in 14% of patients (95% CI 12% to 16%), either as persisting IM after treatment (n=78) or recurrent IM during FU (n=72). During median 3 endoscopies^2–4^ after the first IM finding, IM was reproduced in 33% of patients (95% CI 26% to 42%) during one (n=11) or more (n=32) endoscopies (table 4). Three patients (2% (95% CI 0% to 7%)) subsequently developed LGD: 2 developed LGD in the cardia without visible BE 12 months after IM was found and 1 developed a BE island in the tubular oesophagus with LGD 36 months after IM was noted in the cardia. None of the patients with IM progressed to HGD or EAC. In multivariate cox analysis adjusted for potential confounders (age, gender, BE length, worst baseline histology, reflux stenosis, incident lesion), no statistically significant association was found between a finding of non-dysplastic IM in the cardia and the risk for recurrence (adjusted HR 0.5 (95% CI 0.2 to 1.7)).

Random biopsies from the cardia were noted to contain LGD in 9 patients (9/11121; 0.8%) and 23 endoscopies (20/2733; 0.9%) (all defined as ‘recurrences’, table 3). A single patient underwent additional RFA, while the other eight underwent surveillance and during median 2 years FU with two endoscopies, none of these patients progressed to HGD/EAC.

In 2016, we stopped obtaining random cardia biopsies. Outcomes before 2016 (annual recurrence risk 1.0 (95% CI 0.6 to 1.5)) did not differ significantly from those after 2016 (annual recurrence risk 1.0 (95% CI 0.6 to 1.5) (p 0.96)).

### All-cause mortality

During a median FU for vital status of 60 months (p25–p75 38–86) after baseline or 49 months (p25–p75 26–72) after the last treatment, 96 patients died, of which 92 due to unrelated causes (8.0% (6.5% to 9.7%)) and 4 due to metastasised EAC (0.3% (0.0% to 0.7%)). Most common causes of death were neoplasms other than EAC (35/92; 38%), followed by cardiovascular disease (24/93; 26%) and respiratory disease (13/93; 14%). Online supplemental figure S6 shows the cumulative incidence of unrelated death and recurrence during FU.

10.1136/gutjnl-2020-322615.supp7 Supplementary data

### Progression to EAC exceeding the boundaries for endoscopic treatment

Overall, 22 patients (22/1386; 1.6% (95% CI 1.1% to 2.4%)) progressed to disease that exceeded guideline boundaries for curative endoscopic treatment, either during treatment (n=17) or during FU after CE-BE was established (n=5). The individual case histories of all these 22 patients are presented in online supplemental table S4.

## Discussion

We report treatment outcomes and long-term FU for all 1386 patients with BE-related neoplasia (ie, LGD, HGD and low-risk EAC) who underwent endoscopic treatment with RFA since 2008, based on a nationwide cohort with treatment provided exclusively in expert centres. Treatment was effective in eradicating all BE in 94% of patients. Most failures had achieved a complete eradication of HGD/EAC, yet 1% of patients progressed to disease stages that exceeded the boundaries for curative endoscopic treatment. The majority of these patients (68%) underwent curative surgery or was unfit for major surgery and had died of unrelated causes or was alive at the moment of data collection.

Long-term effects were analysed over median 43 months with median 4 endoscopies and showed sustained eradication of dysplasia in 97%. The majority of the recurrences underwent curative endoscopic treatment, yet only 0.4% of all patients progressed to advanced EAC. Frequent FU visits in the first year did not contribute to detection of recurrences, nor did random biopsies from NSE or the cardia. Our data suggest that in expert centres, FU intervals after CE-BE may be extended, 3-monthly endoscopies in the first year may be omitted and random biopsies from NSE and cardia abandoned.

Successful treatment has previously been reported in 74%–98% of patients^4–7^ with subsequent annual dysplastic recurrence risks of 1%–20% per patient year,^4 8–13^ in varying cohort studies and registries from USA and Europe. Our outcome for CE-BE (94%) lies at the upper end of this spectrum and our annual recurrence risk at the lower end (1%). Our beneficial rates might partially be explained by the stringent quality control in our study: treatment was only performed in expert centres with dedicated pathologists and endoscopists who had participated in joint training programmes. Baseline ER for visible lesions was performed in 53% of HGD patients (ie, 47% of HGD patients had flat BE with HGD in random biopsies and underwent RFA) and 99% of patients with EAC, as compared with 47% and 77%, respectively, of patients in the UK cohort.^33^ An important difference with RFA studies from the USA is that we incorporated ablation of the gastro-oesophageal junction during each focal RFA procedure, to guarantee optimal treatment of this area.^6 34 35^ In addition, our treatment protocol allowed for additional, low-threshold touch-up ER or APC for remaining BE islands after RFA and for additional focal RFA for persisting IM in the GEJ post-RFA. Finally, persisting IM in a normal appearing GEJ after treatment was included in our definition for success, and treatment success was assessed during a single endoscopy.

The stenosis rate of 15% is relatively high as compared with other studies.^4 6 33^ Most prospective clinical trials have restrictions in BE length (ie, less than 8–10 cm) and in extent of prior ER (ie, <2 cm in length and/or <50% of the circumference). However, in current registry, we included all patients independent of BE length or extent of ER. Since these factors had an association with stenosis in our analysis, this may have contributed to the high stenosis rate in the current study.

Our data stress the importance of careful inspection prior to each RFA treatment. Although baseline ER was performed for 62% of patients, incident lesions were found in 5% of patients after RFA was initiated. All patients who progressed to advanced disease were identified as an incident lesion. If visible abnormalities are not recognised and removed with ER but inadvertently treated with RFA, this may lead to incomplete treatment, resulting in progression that remains undetected during the treatment course. Such occurrence may place the patient outside the window of opportunity for curative endoscopic treatment and even for curative surgery.

Although the majority of patients with an incident lesion had curative ER, 10% had progressed to high-risk cancer and required esophagectomy. Overall, 1% of patients had progressed to advanced EAC that exceeded boundaries for curative endoscopic treatment. The majority of progressors were identified at baseline as ‘complicated’ cases with BE neoplasia, due to ultralong BE segments, multifocal neoplasia and/or severe reflux disease. Extra caution is therefore recommended for these patients.

Our data show that post-RFA recurrences are rare. The annual incidence was 1% for recurrent LGD/HGD/EAC and 0.8% for recurrent HGD/EAC, which indicates that if one would follow 200 patients for 5 year, only 8 will develop HGD/EAC. These rates are comparable to a non-dysplastic BE population under endoscopic surveillance, where FU is performed every 3–5 years.^1^

Prior FU studies suggested that most recurrences occurred in the first year after treatment^12 36^ and guidelines therefore suggest to perform 3-monthly endoscopies during the first year of FU, identical to the preablation era when visible lesions were removed with ER and the remaining flat BE was left untreated. The aforementioned studies included recurrent non-dysplastic BE and even IM in the cardia in the definition of recurrence. In our study, we also found more non-dysplastic BE, specifically diminutive islands, during the first 2 years after treatment as compared with the years thereafter. In our opinion, these small areas of non-dysplastic BE could very likely be *residual* tiny BE islands rather than *recurrent* BE. Either way, these small islands were easily treated with a single APC treatment and were found to be of low clinical relevance. In our opinion, these findings do therefore not justify more frequent FU visits.

Our cohort could be considered as a natural experiment for the effects of 3-monthly endoscopies during the first year of FU. Until 2014, this frequent FU schedule was default while FU was performed on an annual basis from 2015 onwards. Although the cumulative incidence of LGD/HGD/EAC was slightly higher in the patients with 3-monhtly endoscopies FU (2.8 vs 1.4%), this difference was not statistically significant and was mainly based on an increased detection of LGD in the cardia, which given the absence of progression to HGD/EAC during FU, was of dubious clinical relevance. Since the reason to perform frequent FU is to prevent progression to advanced neoplasia, this should be the most important outcome in our natural experiment. Although numbers are very low, the risk appeared comparable (0.7% for frequent FU and 0.4% for annual FU, P0.4). Overall, our data suggest that frequent FU in the first year is not associated with clinically relevant recurrence during FU and can be loosened.

Furthermore, background mortality is significant in the post-treatment BE population and we recommend that this may be taken into account when defining of the need for and frequency of post-RFA FU. We are currently developing an evidence based post-RFA FU regimen based on a balance between the risk for recurrent, clinically relevant Barrett’s neoplasia and a patient’s overall life expectancy and risk to die of other causes.

Our data suggest that there is no need for random biopsies in post-RFA FU when treatment is performed in expert centres. All HGD/EAC recurrences appeared as endoscopically visible abnormalities and none were detected through random biopsies alone. Careful inspection of the NSE along the length of the initial BE, with targeted biopsies of any visible abnormality, is therefore the most essential part of FU. Crucial part of the inspection is careful retroflexed inspection of the cardia, since 18% of the HGD/EAC recurrences in this study occurred in this area, and these can easily be overlooked during inspection with the endoscope in antegrade position.

NSE random biopsies showed buried BE in 3% of patients, a finding that was neither reproduced nor associated with neoplastic progression during median 4 years of FU and a median of 4 endoscopies. Our findings are in line with other studies^12^ and supports our decision to change in our FU strategy in 2013 by abandoning random NSE biopsies.

Cardia random biopsies were noted to contain IM in 14% of patients or 7% of endoscopies. Our data suggest that this is no clinically relevant disease and no indication for treatment: during median 3 years of FU with a median of 3 endoscopies, the finding was reproduced in only 33% of the patients and none progressed to neoplasia. This is in line with prior studies that showed reproduction of IM in 11%–33% during median 3–5 FU endoscopies.^4 9 37^ A recent study showed no increased risk for dysplasia among patients with recurrent IM of the cardia.^38^ These outcomes are comparable to those reported for a healthy, asymptomatic population without BE. IM can occur in 4%–15% of the normal population,^39–44^ and a study from the Mayo Clinic followed 86 patients with a diagnosis of IM of the cardia for 8 years, during which none progressed to neoplasia.^45^ Accordingly, Krajciova *et al* showed in their retrospective analysis of 136 patients with successful ablation, that persisting IM after treated or recurrent IM during FU, detected in random biopsies from a normal appearing cardia, was not associated with an increased risk for dysplastic recurrence.^45^

Apart from IM, an endoscopically normal cardia was found to contain LGD in 0.8% of patients or 0.7% of endoscopies. Although we defined this as a recurrence, the clinical relevance of this finding was negligible. None of the patients progressed to HGD/EAC and this is in line with the aforementioned study from the Mayo Clinic, which showed no progression in eight patients with LGD of the cardia.^45^ Since we have stopped obtaining random biopsies from the cardia in 2016, this entity of ‘invisible’ LGD in the cardia will no longer be detected and, based on the low risk for progression, this appears justified. Moreover, since patients are kept under endoscopic surveillance, potential progression to HGD or worse may still be identified and treated at early stages.

Long-term endpoints for treatment of BE neoplasia have undergone significant transformation over the years. Initially, esophagectomy was the standard therapy and success was defined as 5-year tumor-free survival. Currently, endoscopic treatment is treatment of choice and given the extremely low mortality rates, EAC-related death has no longer been an appropriate endpoint. Instead, increasingly more stringent definitions have been used over time and nowadays, some studies report sustained eradication of all BE including invisible IM in the cardia.^12^ Although a complete eradication of BE reflects an appropriate *treatment outcome* for RFA, it does not express the outcome of interest *during FU*. CE-BE after treatment may in fact be considered as an intermediate endpoint for the outcome of interest and the main motive to initiate RFA, that is, a reduction in the risk for future (advanced) neoplasia. Therefore, we suggest that recurrent neoplasia and not BE or IM should be the primary endpoint for assessment of long-term outcomes.

This study has important strengths. This is the first report of a nationwide cohort of patients with BE with long-term FU after centralised treatment in expert centres. Our data are homogeneous: all endoscopists and pathologists participated in a specific and joint training programme and all centres followed a uniform treatment and FU protocol. We included all patients in the Netherlands who underwent EET. We provide high-quality data that were collected by dedicated researchers and with central discussion of all patients with endpoints. A rigorous treatment and FU protocol in all BECs and meticulous data collection resulted in only 1% of our patients that were lost to FU.

We have to address some limitations as well. Although our patients were registered prospectively, most of the actual data collection was done retrospectively with a risk for bias, specifically selection and information bias. All patients in the current study underwent at least one RFA treatment and results are therefore only applicable to patients undergoing RFA treatment. As shown in figure 1, 94 patients underwent ER monotherapy with surveillance of the remaining BE instead of RFA. Although in a majority of patients RFA was not initiated due to limited life expectancy, this decision may have (partially) been based on expected poor response after RFA, for example, due to BE regeneration of the ER wound. Long-term outcomes of these 94 patients have been described separately.^27^ During median 21 months FU with 4 endoscopies per patient, 17 patients (18%) progressed to HGD/EAC. No patient progressed to advanced EAC. Endoscopic surveillance of a remaining BE segment after ER, instead of RFA, may be the preferred treatment strategy in selected patients.

Furthermore, 27% of our patients had LGD at baseline, and comparisons with HGD/EAC cohorts should therefore be made with caution. Information bias may have been present due to data collection by different persons, although random checks were performed by a second person for 50% of patients. Still, we had only few missing data due to standardised endoscopy and pathology reports in all centres. Furthermore, the assessment whether the cardia appears abnormal or normal, and thus whether biopsies should be obtained or not, may be operator dependent. Our study included only patients in the Netherlands, which limits the generalisability. All patients underwent endoscopic workup and treatment at expert centres and the results of this study can therefore not automatically be extrapolated to general practice. Current guidelines however recommend centralisation of EET for patients with Barrett’s neoplasia in dedicated centres with multidisciplinary experience in this field (ie, experience in endoscopic imaging and treatment, sufficient case volumes, expert GI-pathology, and access to oesophageal surgery). Finally, although all centres followed the central treatment protocol that advised on which regimen should be used, we have no data on RFA regimen.

In conclusion, this large cohort of all Dutch patients treated with RFA±ER for BE with dysplasia or low-risk EAC, according to a uniform treatment protocol in a centralised setting, demonstrates that this approach successfully eradicates the BE segment in 94% of patients. Post-RFA recurrences are rare. Clinically relevant recurrences are detected as endoscopic abnormalities and at stages generally amendable for curative endoscopic treatment. Our data suggest that post-RFA FU can be simplified: we may abandon 3-monthly endoscopies in the first year of FU and we may stop random sampling of NSE and cardia. Instead, dedicated endoscopic inspection, and if needed target biopsies are the most important steps to detect post-RFA recurrences.

10.1136/gutjnl-2020-322615.supp3 Supplementary data

## Data Availability

Data are available upon reasonable request. Additional information (protocols, statistical analysis) are available upon request.

## References

[1] Weusten B , Bisschops R , Coron E , et al . Endoscopic management of Barrett's esophagus: European Society of gastrointestinal endoscopy (ESGE) position statement. Endoscopy 2017;49:191–8. 10.1055/s-0042-122140 28122386

[2] Gastroenterologists DSo . Richtlijn Barrett-Oesofagus, 2017.

[3] Sharma P , Shaheen NJ , Katzka D , et al . AGA clinical practice update on endoscopic treatment of Barrett's esophagus with dysplasia and/or early cancer: expert review. Gastroenterology 2020;158:760–9. 10.1053/j.gastro.2019.09.051 31730766

[4] Phoa KN , Pouw RE , Bisschops R , et al . Multimodality endoscopic eradication for neoplastic Barrett oesophagus: results of an European multicentre study (EURO-II). Gut 2016;65:555–62. 10.1136/gutjnl-2015-309298 25731874

[5] Phoa KN , van Vilsteren FGI , Weusten BLAM , et al . Radiofrequency ablation vs endoscopic surveillance for patients with Barrett esophagus and low-grade dysplasia: a randomized clinical trial. JAMA 2014;311:1209–17. 10.1001/jama.2014.2511 24668102

[6] Shaheen NJ , Sharma P , Overholt BF , et al . Radiofrequency ablation in Barrett's esophagus with dysplasia. N Engl J Med 2009;360:2277–88. 10.1056/NEJMoa0808145 19474425

[7] Desai M , Saligram S , Gupta N , et al . Efficacy and safety outcomes of multimodal endoscopic eradication therapy in Barrett's esophagus-related neoplasia: a systematic review and pooled analysis. Gastrointest Endosc 2017;85:482–95. 10.1016/j.gie.2016.09.022 27670227

[8] Wani S , Puli SR , Shaheen NJ , et al . Esophageal adenocarcinoma in Barrett's esophagus after endoscopic ablative therapy: a meta-analysis and systematic review. Am J Gastroenterol 2009;104:502–13. 10.1038/ajg.2008.31 19174812

[9] Phoa KN , Pouw RE , van Vilsteren FGI , et al . Remission of Barrett's esophagus with early neoplasia 5 years after radiofrequency ablation with endoscopic resection: a Netherlands cohort study. Gastroenterology 2013;145:96–104. 10.1053/j.gastro.2013.03.046 23542068

[10] Pasricha S , Bulsiewicz WJ , Hathorn KE , et al . Durability and predictors of successful radiofrequency ablation for Barrett's esophagus. Clin Gastroenterol Hepatol 2014;12:1840–7. 10.1016/j.cgh.2014.04.034 24815329 PMC4225183

[11] Cotton CC , Haidry R , Thrift AP , et al . Development of evidence-based surveillance intervals after radiofrequency ablation of Barrett's esophagus. Gastroenterology 2018;155:316–26. 10.1053/j.gastro.2018.04.011 29655833 PMC6067977

[12] Sami SS , Ravindran A , Kahn A , et al . Timeline and location of recurrence following successful ablation in Barrett's oesophagus: an international multicentre study. Gut 2019;68:1379–85. 10.1136/gutjnl-2018-317513 30635408

[13] Pouw RE , Klaver E , Phoa KN , et al . Radiofrequency ablation for low-grade dysplasia in Barrett’s esophagus: long-term outcome of a randomized trial. Gastrointest Endosc 2020. 10.1016/j.gie.2020.03.3756 32217112

[14] Curvers WL , ten Kate FJ , Krishnadath KK , et al . Low-Grade dysplasia in Barrett's esophagus: overdiagnosed and underestimated. Am J Gastroenterol 2010;105:1523–30. 10.1038/ajg.2010.171 20461069

[15] Duits LC , Phoa KN , Curvers WL , et al . Barrett's oesophagus patients with low-grade dysplasia can be accurately risk-stratified after histological review by an expert pathology panel. Gut 2015;64:700–6. 10.1136/gutjnl-2014-307278 25034523

[16] Duits LC , van der Wel MJ , Cotton CC , et al . Patients With Barrett's Esophagus and Confirmed Persistent Low-Grade Dysplasia Are at Increased Risk for Progression to Neoplasia. Gastroenterology 2017;152:993–1001. 10.1053/j.gastro.2016.12.008 28012849

[17] van der Wel MJ , Klaver E , Duits LC , et al . Adherence to pre-set benchmark quality criteria to qualify as expert assessor of dysplasia in Barrett's esophagus biopsies - towards digital review of Barrett's esophagus. United European Gastroenterol J 2019;7:889–96. 10.1177/2050640619853441 PMC6683647 31428413

[18] Bergman JJGHM , de Groof AJ , Pech O , et al . An interactive web-based educational tool improves detection and delineation of Barrett's Esophagus-Related neoplasia. Gastroenterology 2019;156:1299–308. 10.1053/j.gastro.2018.12.021 30610858

[19] Curvers WL , van Vilsteren FG , Baak LC , et al . Endoscopic trimodal imaging versus standard video endoscopy for detection of early Barrett's neoplasia: a multicenter, randomized, crossover study in general practice. Gastrointest Endosc 2011;73:195–203. 10.1016/j.gie.2010.10.014 21168835

[20] de Groof J , van der Sommen F , van der Putten J , et al . The argos project: the development of a computer-aided detection system to improve detection of Barrett's neoplasia on white light endoscopy. United European Gastroenterol J 2019;7:538–47. 10.1177/2050640619837443 PMC6488793 31065371

[21] Barret M , Belghazi K , Weusten BLAM , et al . Single-session endoscopic resection and focal radiofrequency ablation for short-segment Barrett's esophagus with early neoplasia. Gastrointest Endosc 2016;84:29–36. 10.1016/j.gie.2015.12.034 26769410

[22] Belghazi K , Marcon N , Teshima C , et al . Risk factors for serious adverse events associated with multiband mucosectomy in Barrett's esophagus: an international multicenter analysis of 3827 endoscopic resection procedures. Gastrointest Endosc 2020;92:259–68. 10.1016/j.gie.2020.03.3842 32240684

[23] Belghazi K , Pouw RE , Koch AD , et al . Self-sizing radiofrequency ablation balloon for eradication of Barrett's esophagus: results of an international multicenter randomized trial comparing 3 different treatment regimens. Gastrointest Endosc 2019;90:415–23. 10.1016/j.gie.2019.05.023 31108093

[24] Pouw RE , Künzli HT , Bisschops R , et al . Simplified versus standard regimen for focal radiofrequency ablation of dysplastic Barrett's oesophagus: a multicentre randomised controlled trial. Lancet Gastroenterol Hepatol 2018;3:566–74. 10.1016/S2468-1253(18)30157-2 29934224

[25] Pouw RE , van Vilsteren FGI , Peters FP , et al . Randomized trial on endoscopic resection-cap versus multiband mucosectomy for piecemeal endoscopic resection of early Barrett's neoplasia. Gastrointest Endosc 2011;74:35–43. 10.1016/j.gie.2011.03.1243 21704807

[26] van Vilsteren FGI , Alvarez Herrero L , Pouw RE , et al . Predictive factors for initial treatment response after circumferential radiofrequency ablation for Barrett's esophagus with early neoplasia: a prospective multicenter study. Endoscopy 2013;45:516–25. 10.1055/s-0032-1326423 23580412

[27] van Munster SN , Nieuwenhuis EA , Weusten BLAM , et al . Endoscopic resection without subsequent ablation therapy for early Barrett's neoplasia: endoscopic findings and long-term mortality. J Gastrointest Surg 2021;25:67–76. 10.1007/s11605-020-04836-8 33140322 PMC7851009

[28] Künzli HT , Schölvinck DW , Phoa KN , et al . Simplified protocol for focal radiofrequency ablation using the HALO90 device: short-term efficacy and safety in patients with dysplastic Barrett's esophagus. Endoscopy 2015;47:592–7. 10.1055/s-0034-1391436 25675174

[29] Alvarez Herrero L , van Vilsteren FGI , Pouw RE , et al . Endoscopic radiofrequency ablation combined with endoscopic resection for early neoplasia in Barrett's esophagus longer than 10 cm. Gastrointest Endosc 2011;73:682–90. 10.1016/j.gie.2010.11.016 21292262

[30] Alvarez Herrero L , Curvers WL , van Vilsteren FGI , et al . Validation of the Prague C&M classification of Barrett's esophagus in clinical practice. Endoscopy 2013;45:876–82. 10.1055/s-0033-1344952 24165812

[31] Pouw RE , Visser M , Odze RD , et al . Pseudo-buried Barrett's post radiofrequency ablation for Barrett's esophagus, with or without prior endoscopic resection. Endoscopy 2014;46:105–9. 10.1055/s-0033-1358883 24285123

[32] van der Wel MJ , Duits LC , Klaver E , et al . Development of benchmark quality criteria for assessing whole-endoscopy Barrett's esophagus biopsy cases. United European Gastroenterol J 2018;6:830–7. 10.1177/2050640618764710 PMC6047285 30023060

[33] Haidry RJ , Lipman G , Banks MR , et al . Comparing outcome of radiofrequency ablation in Barrett's with high grade dysplasia and intramucosal carcinoma: a prospective multicenter UK registry. Endoscopy 2015;47:980–7. 10.1055/s-0034-1392414 26126159

[34] Lyday WD , Corbett FS , Kuperman DA , et al . Radiofrequency ablation of Barrett's esophagus: outcomes of 429 patients from a multicenter community practice registry. Endoscopy 2010;42:272–8. 10.1055/s-0029-1243883 20146164

[35] Fleischer DE , Overholt BF , Sharma VK , et al . Endoscopic radiofrequency ablation for Barrett's esophagus: 5-year outcomes from a prospective multicenter trial. Endoscopy 2010;42:781–9. 10.1055/s-0030-1255779 20857372

[36] Vliebergh JH , Deprez PH , de Looze D , et al . Efficacy and safety of radiofrequency ablation of Barrett's esophagus in the absence of reimbursement: a multicenter prospective Belgian registry. Endoscopy 2019;51:317–25. 10.1055/a-0739-7679 30360011

[37] Pouw RE , Klaver E , Phoa KN , et al . Radiofrequency ablation for low-grade dysplasia in Barrett's esophagus: long-term outcome of a randomized trial. Gastrointest Endosc 2020;92:569–74. 10.1016/j.gie.2020.03.3756 32217112

[38] Solfisburg QS , Sami SS , Gabre J , et al . Clinical significance of recurrent gastroesophageal junction intestinal metaplasia after endoscopic eradication of Barrett's esophagus. Gastrointest Endosc 2020. 10.1016/j.gie.2020.10.027. [Epub ahead of print: 02 Nov 2020]. 33144238

[39] Siddiki HA , Lam-Himlin DM , Kahn A , et al . Intestinal metaplasia of the gastric cardia: findings in patients with versus without Barrett's esophagus. Gastrointest Endosc 2019;89:759–68. 10.1016/j.gie.2018.10.048 30447215

[40] Byrne JP , Bhatnagar S , Hamid B , et al . Comparative study of intestinal metaplasia and mucin staining at the cardia and esophagogastric junction in 225 symptomatic patients presenting for diagnostic open-access gastroscopy. Am J Gastroenterol 1999;94:98–103. 10.1111/j.1572-0241.1999.00778.x 9934738

[41] Zaninotto G , Avellini C , Barbazza R , et al . Prevalence of intestinal metaplasia in the distal oesophagus, oesophagogastric junction and gastric cardia in symptomatic patients in north-east Italy: a prospective, descriptive survey. The Italian Ulcer Study Group "GISU". Dig Liver Dis 2001;33:316–21. 10.1016/s1590-8658(01)80084-0 11432508

[42] Spechler SJ , Zeroogian JM , Antonioli DA , et al . Prevalence of metaplasia at the gastro-oesophageal junction. Lancet 1994;344:1533–6. 10.1016/s0140-6736(94)90349-2 7983953

[43] Peck-Radosavljevic M , Püspök A , Pötzi R , et al . Histological findings after routine biopsy at the gastro-oesophageal junction. Eur J Gastroenterol Hepatol 1999;11:1265–70. 10.1097/00042737-199911000-00014 10563538

[44] Hirota WK , Loughney TM , Lazas DJ , et al . Specialized intestinal metaplasia, dysplasia, and cancer of the esophagus and esophagogastric junction: prevalence and clinical data. Gastroenterology 1999;116:277–85. 10.1016/s0016-5085(99)70123-x 9922307

[45] Jung KW , Talley NJ , Romero Y , et al . Epidemiology and natural history of intestinal metaplasia of the gastroesophageal junction and Barrett's esophagus: a population-based study. Am J Gastroenterol 2011;106:1447–55. 10.1038/ajg.2011.130 21483461 PMC3150349

