# Supplementary material for: Long-term outcomes after endoscopic treatment for Barrett’s neoplasia with radiofrequency ablation ± endoscopic resection: results from the national Dutch database in a 10-year period
Source: Gut. 2021 Mar 22;71(2):265–76. doi: 10.1136/gutjnl-2020-322615 (PMC8762001; doi:10.1136/gutjnl-2020-322615)
Supplement: Supplementary data [file gutjnl-2020-322615supp001.pdf]

Supplementary material

Tables

Table S1. Qualifications for a Barrett Expert Center

Table S2. Definitions

Table S3. Treatment success and failures

Table S4. Progression to advanced disease

Table S5. Risk factors for esophageal stenosis

Table S6. Recurrent NDBE

Table S7. Yield of 3-monthly endoscopies in the first year after CE-BE

Figures

Figure S1. Changes in outcomes over time

Figure S2. Three types of recurrent BE neoplasia during FU

Figure S3. Timing and location of recurrences

Figure S4. Timing of recurrent non-dysplastic BE tongues and BE islands

Figure S5. Recurrent non-dysplastic BE tongues and islands

Figure S6. Risk for recurrence and unrelated death during long-term FU

Table S1. Qualifications for a Barrett Expert Center

Annual case load for new BE dysplasia >10
1-2 dedicated endoscopists with joint training and demonstrable further education[1]
1-2 dedicated pathologists with joint training and demonstrable further education[2]
High-resolution endoscopic equipment
Expertise to handle complications with access to surgical expertise
Multidisciplinary consultation (surgery, oncology, pathology) for patients with BE neoplasia
Participation in quarterly meetings and case discussions
Adherence to the joint treatment/FU protocol
Prospective registration of all patients in a database

Qualifications for a center to be a Barrett Expert Center, according to the Dutch Barrett guideline (2).

1: Endoscopists all participated in quarterly meetings with discussion of difficult cases, discussion of new clinical studies and recent literature.

2: All pathologists assessed a digitalized set of 60 whole endoscopy slides, followed by 80 digital cases with 2 consensus meetings; pathologists participated in bi-annual group meetings to discuss difficult cases and recent literature.

Table S2. Definitions

Term	Definition
Advanced EAC exceeding the boundaries for curative endoscopic treatment	BE neoplasia exceeding the boundaries for curative endoscopic treatment, due to high-risk EAC (≥sm2-EAC, poor differentiation, lymphovascular invasion, or irradical vertical resection margin) or neoplasia that based on its endoscopic appearance as a visible lesion would require an endoscopic resection yet which could technically not be performed based on extent or local scarring.
BE related dysplasia and early cancer (treatment indication)	BE containing LGD, HGD, or low-risk EAC (defined as ≤sm1-EAC with good-moderate differentiation, no lymphovascular invasion, and radical vertical resection margin)
Bleeding	Bleedings (signs of hematemesis, melena, or drop in hemoglobin level) that occurred after the endoscopy and for which a hospital admission or a medical intervention was required.
Complete endoscopic eradication of BE (CE-BE)	Complete eradication of all visible Barrett mucosa and all dysplasia. Patients with complete endoscopic eradication of all visible BE, yet persisting IM in the random cardia biopsies, were considered CE-BE.
Complication, fatal	Death attributable to procedure <30 days or longer with continuous hospitalization
Complication, mild	Unplanned hospital admission, hospitalization < 3 days, haemoglobin drop <3 g, no transfusion
Complication, moderate	4–10 days hospitalisation, <4 units blood transfusion, repeat endoscopic intervention, radiological intervention
Complication, severe	hospitalisation >10 days, intensive care unit (ICU) admission, need for surgery, >4 units blood transfusion, in the case of stenosis: >5 dilatations, stent placement or incision therapy
Failure, real	Failure for CE-BE with >20% of the initial BE remaining and/or persisting neoplasia
Failure, elective decision	Failure for CE-BE with <20% of the initial BE remaining and no neoplasia, in whom an elective decision was made to withhold further treatment due to expected minimal benefits from further therapy
Perforation	Transmural defect of esophageal wall during or immediately following the endoscopy, and/or free air or leakage on radiologic examination
Per-protocol population	All patients in the RFA treatment cohort, who completed the treatment protocol. Patients were excluded for this analysis, if A) unrelated death occurred during treatment, or B) a significant change in comorbidity occurred during treatment and continued RFA was considered medically unjustified.
Poor healing	Visible ulcerations ≥3 months after RFA treatment
Poor squamous regeneration	<50% squamous regression after RFA treatment
Recurrent non-dysplastic BE	Recurrent endoscopically visible BE in the tubular esophagus, with random biopsies showing IM without dysplasia
Severe reflux esophagitis	Los Angeles Classification Grade C/D reflux esophagitis
Stenosis	(a)Symptomatic esophageal narrowing requiring an intervention (e.g. endoscopic dilatation, incision therapy, or stent placement).
Sustained eradication of dysplasia	Complete and sustained eradication of LGD, HGD, or EAC during long-term endoscopic follow-up. A patient was considered a failure for this endpoint if recurrent LGD, HGD, or EAC was detected in the tubular esophagus or cardia, or if lymph node or distant metastasis from EAC were found during follow-up.
Sustained eradication of HGD/EAC	Complete and sustained eradication of HGD, or EAC during long-term endoscopic follow-up. A patient was considered a failure for this endpoint if recurrent HGD, or EAC was detected in any of the biopsies or ER specimen from the tubular esophagus or cardia, or if lymph node or distant metastasis from EAC were found during follow-up.
Touch-up treatment	Any residual BE persisting after RFA sessions could be treated with a single ER session (for areas >5 mm) or with a maximum of two argon plasma coagulation (APC) sessions in case of areas <5 mm.

Treatment failure	Patients were considered a failure for CE-BE if residual visible BE persisted after completing the treatment protocol, including –when necessary– a single ER or a maximum of two touch-up APC treatments, and/or if residual dysplasia persisted in biopsies from the cardia.
Visible lesion	Any mucosal irregularity or discoloration within the BE

Table S3. Treatment success and failures

	Succes	Treatment failures		
	CE-BE<br><br>N=1270	All<br><br>N=78	Elective treatment stop<br>N=34	Real treatment failures<br>N=44
Age, years, mean (±sd)	64 (9)	68 (9)	70 (10)	66 (8)
Male gender, n (%)	1039 (82)	55 (70)	23 (68)	32 (72)
Initial BE length, cm, median (IQR)	C2M4 (0-5; 3-7)	C8M9 (4-10; 6-12)	C4M6 (3-8; 4-9)	C10M11 (6-12; 7-13)
Initial histology, n (%)<br>LGD<br>HGD<br>EAC	 350 (28)<br>391 (31)<br>529 (42)	 16 (21)<br>17 (22)<br>45 (58)	 7 (21)<br>7 (21)<br>20 (59)	 9 (21)<br>10 (23)<br>25 (57)
ER, n (%)	781 (62)	56 (72)	24 (73)	32 (73)
C-RFA, N, median (IQR)	1 (0-1)	1 (1-1)	1 (0-1)	1 (1-2)
F-RFA, N, median (IQR)	2 (1-2)	1 (0-2)	1 (1-2)	0 (0-1)
Duration of treatment (months, IQR)	10 (5-13)	10 (5-22)	14 (7-29)	8 (3-15)
Persisting IM in normal appearing GEJ, n (%)	85 (7)			
Extent of residual BE, cm, median (IQR)		C1M4 (0-6; 2-8)	C0M2 (0-1; 1-2)	C5M7 (2-7; 5-11)
Reason to stop, n (%)<br>High-risk EAC<br>Multifocal lesions<br>Poor squamous regeneration<br>Esophageal stenosis<br>All BE and HGD/EAC eradicated**		 7 (9)<br>10 (13)<br>38 (49)<br>14 (18)<br>9 (12)	 -<br>-<br>11 (32)<br>14 (41)<br>9 (26)	 7 (16)<br>10 (23)<br>27 (61)<br>-<br>-
Residual PA, n (%)<br>NDBE/LGD<br>HGD/EAC		 58 (74)<br>20 (26)	 34 (100)<br>-	 24 (55)<br>20 (45)
Final outcome, n (%)<br>Progression*<br>CE-BE after extensive ER<br>Endoscopic surveillance<br>No further surveillance	 -<br>-<br>1,154***	 17 (22)<br>5 (6)<br>52 (67)<br>4 (5)	 -<br>2 (6)<br>29 (85)<br>3 (9)	 17 (39)<br>3 (7)<br>23 (53)<br>1 (2)
Endoscopic surveillance<br>Duration, mo, mean (±sd)<br>Endoscopies, n, mean (±sd)<br>HGD/EAC, n(%)	 39 (29)<br>3 (2)<br>24 (2)	 47 (31)<br>4 (3)<br>13 (25)	 49 (22)<br>4 (3)<br>6 (18)	 45 (39)<br>5 (4)<br>7 (30)

Abbreviations: BE – Barrett's esophagus; C-RFA – circumferential RFA; CE-BE – complete endoscopic eradication of Barrett's esophagus; EAC – esophageal adenocarcinoma; ER – endoscopic resection; F-RFA – focal RFA; GEJ – gastroesophageal junction; HGD – high-grade dysplasia; IQR – interquartile range; LGD – low-grade dysplasia; mo – months; NDBE – non-dysplastic BE; SD – standard deviation

*Disease progression to ta stage that exceeded boundaries for curative endoscopic treatment
**Only persisting LGD in the cardia
*** 1,154 patients with CE-BE and endoscopic surveillance afterwards

Table S4. Progression to advanced neoplasia

S5A. Progression during treatment phase													
Pt nr	Treatment year	Initial BE (cm)	Reflux -itis a/o stenosis	Lesions (n)	Baseline histology	ER (n pieces)	RFA (n), (C/F)	PH, PSR	Incident lesion, treatment (histology)	Time (mo)	Indication for surgery	Esophagectomy	Outcome
	Progression to EAC exceeding the boundaries for curative endoscopic treatment												
1	2011	C15M15	No	1	M3-EAC	Yes (4)	5 (3/2)	Yes	Yes, ER (sm,R1)	32	PSR with progression to high-risk EAC	Yes (T2N1M0)	Curative surgery, unrelated death
2	2009	C8M10	No	1	M3-EAC, residual HGD	Yes (4)	2 (0/2)	No	Yes, ER (sm, G3, R1)	13	Progression to high-risk EAC	Yes (T1bN2M0)	Curative surgery, unrelated death
3	2017	C1M2	No	1	M3-EAC	Yes (2)	1 (0/1)	No	Yes, ER (sm2, G3, R1)	10	Progression to high-risk EAC	Yes (T2N0M0)	Curative surgery, alive
4	2011	C5M7	Yes	2	M3-EAC, residual LGD	Yes (2)	2 (1/1)	Yes	Yes, ER (sm1, LVI+, R1)	10	PSR with progression to high-risk EAC	Yes (TisN0M0)	Curative surgery, alive
5	2009	C5M8	No	-	Multifocal HGD	No	3 (1/2)	No	Yes, ER (sm2, LVI+, R1)	22	Progression to high-risk EAC	Yes (T1bN0M0)	Curative surgery, alive
6	2012	C11M14	Yes	2	HGD, residual HGD	Yes (4)	1 (1/0)	Yes	Yes, ER (sm, R1)	19	PSR with progression to high-risk EAC	No, patient refused surgery	EAC-related death
7	2009	C15M15	Yes	-	Multifocal HGD	No	2 (2/0)	Yes	Yes, ER (sm, LVI+, R1)	8	PSR with progression to high-risk EAC	No, unfit for surgery	EAC-related death
	Endoscopic resection technically impossible due to multifocality and/or post-treatment fibrosis												
8	2008	C11M13	No	2	M3-EAC, residual EAC	Yes (2)	1 (1/0)	No	Multifocal, no ER (EAC)	8	Multifocal EAC	Yes (T1aN0M0)	Curative surgery, alive
9	2008	C10M12	No	1	M2-EAC	Yes (3)	2 (2/0)	Yes	Multifocal, partial ER (HGD)	9	Multifocal HGD and PSR	Yes (T1aN0M0)	Curative surgery, alive
10	2008	C12M13	No	3	M3-EAC	Yes (7)	1 (1/0)	Yes	Multifocal, no ER (EAC)	4	Multifocal EAC and PSR	Yes (T1aN0M0)	Curative surgery, alive
11	2016	C6M11	No	2	M3-EAC, residual unk	Yes (7)	1 (1/0)	Yes	Multifocal, partial ER (HGD)	22	Multifocal HGD and PSR	Yes (T0N0M0)	Curative surgery, alive
12	2015	C2M6	No	2	M2-EAC, residual HGD	Yes (4)	3 (0/3)	No	Multifocal, partial ER (LGD)	14	Rapidly growing, multifocal abnormalities	No, unfit for surgery	EAC-related death
13	2015	C10M11	Yes	1	M3-EAC, residual unk	Yes (4)	2 (2/0)	Yes	Multifocal, no ER (EAC)	6	Multifocal EAC, PSR, and stenosis	No, unfit for surgery	EAC-related death
14	2012	C11M12	No	1	M3-EAC, residual unk	Yes (1)	2 (2/0)	Yes	Multifocal, no ER (HGD)	10	Multifocal HGD and PSR	No, unfit for surgery	Unrelated death
15	2013	C12M13	No	1	Sm1-EAC	Yes (10)	2 (1/1)	No	Yes, incomplete ER (EAC)	21	Persisting lesion with EAC and severe fibrosis	No, unfit for surgery	Unrelated death
16	2014	C14M16	No	2	M3-EAC, residual HGD	Yes (14)	1 (0/1)	Yes	Multifocal, no ER (HGD)	5	Multifocal HGD and PSR	No, unfit for surgery	Alive

17	2010	C11M13	No	1	M2-EAC, residual unk	Yes (3)	1 (1/0)	Yes	Multifocal, partial ER (EAC)	6	Multifocal EAC and PSR	No, unfit for surgery	Alive

	**S5B. Progression during follow-up**													
Patient	Treartment year	Initial BE (cm)	Reflux -itis a/o stenosis	Lesions (n)	Baseline histology	ER (n pieces)	RFA (n), (C/F)	PH, PSR	Incident lesion, treatment (histology)	Touch-up	FU duration before progression	Type of progression	Esophagectomy	Final outcome
1	2010	C12M13	Yes	2	M3-EAC	Yes (2)	3 (1/2)	No	No	1 APC	30mo, 2 endo	Metastasized EAC, no intra-luminal recurrence	No, metastasized at moment of detection	EAC-related death
2	2008	C8M10	Yes	3	M3-EAC	Yes (2)	2 (2/0)	Yes	No	Multiple APC	24 mo, 2 endo	Recurrent lesion and ER (sm1, G3, R1)	Yes (TxN0M0),	+8 years recurrent EAC in gastric tube, chemoradiation. Alive.
3	2009	C3M5	No	1	M3-EAC	Yes (2)	4 (1/3)	No	No	-	12 mo, 2 endo	Recurrent lesion and ER (sm2, G3, LVI+, R1)	Yes (TxN1M0)	+1.5 years M1, EAC-related death.
4	2011	C8M8	No	-	Multifocal HGD	No	3 (1/2)	No	No	-	30 mo, 2 endo	Recurrent lesion and ER (sm2, G3, LVI+, R0)	Yes (T1bN2M0).	+3 months M1, EAC-related death
5	2016	C10M11	Yes	-	Multifocal HGD	No	2 (1/1)	Yes	Yes, ER (m2-EAC)	1 APC	12 mo, 1 endo	Recurrent lesion and ER/RFA (sm2, R1)	Yes (T1bN1M0)	Curative surgery, alive

Abbreviations: APC – argon plasma coagulation; BE – Barrett's esophagus; C-RFA – circumferential RFA; EAC – esophageal adenocarcinoma; endo – endoscopies; ER – endoscopic resection; F-RFA – focal RFA; FU – follow-up; G3 – poor differentiation; GEJ – gastroesophageal junction; HGD – high-grade dysplasia; IQR – interquartile range; LGD – low-grade dysplasia; LVI – lymphovascular invasion; mo – months; PH – poor healing; PSR – poor squamous regeneration; R1 – irradical resection; SD – standard deviation

Table S5. Risk factors for esophageal stenosis

	No stenosis N = 1176	Stenosis N = 210	P-value
Circumferential extent of BE, cm, median (p25-p75)	2 (0-5)	3 (1-7)	<0.01
Maximum extent of BE, cm, median (p25-p75)	5 (3-7)	6 (4-9)	<0.01
Reflux esophagitis at baseline, n (%)	40 (3)	9 (4)	0.52
Prior ER, n (%)	705 (60)	165 (79)	<0.01
Length of prior ER, mm, median (p25-p75)[1]	20 (15-26)	25 (20-40)	<0.01
Circumferential extent of prior ER, cm, median (p25-p75)[2]	30 (25-50)	37 (24-50)	<0.01
Total number of ER specimen, n, median (p25-p75)[3]	2 (1-3)	3 (2-5)	<0.01

1: 88 were missing
2: 323 were missing
3: 5 were missing

Abbreviations: BE – Barrett's esophagus; ER – endoscopic resection

Table S6. Recurrent NDBE

		**BE tongues N = 27**	**BE islands N = 84**
FU before NDBE	Duration after treatment, mo, median (IQR)	33 (24-48)	15 (11-24)
	N endoscopies after treatment, median (IQR)	3 (2-3)	2 (1-2)
	Patients with IM in cardia before recurrent NDBE, n (%)	3 (11)	4 (5)
Recurrent NDBE	Extent of BE, median (IQR)	C0M2 (0-1; 2-3)	Diminutive islands
Outcomes after NDBE	Treatment, n (%)	5 (19); all RFA	65 (77); all APC
	Surveillance, n (%)	22 (81)	19 (23)
	Duration of surveillance, median (IQR)	20 (10-30)	24 (18-30)
	Progression to LGD, n (%)	1 (5)	0
	Progression to HGD/EAC, n (%)	0	0

Abbreviations: BE – Barrett's esophagus; EAC – esophageal adenocarcinoma; FU – follow-up; HGD – high-grade dysplasia; IM – intestinal metaplasia; IQR – interquartile range; LGD – low-grade dysplasia; mo – months; NDBE – non-dysplastic Barrett's esophagus

Table S7. Yield of frequent FU in the first year of FU

	**Frequent FU (t=0,3,6,9,12mo) N=393**	**Annual FU (t=0, 12mo) N=486**	**P-value**
S6.A Recurrence in the first 30months of FU			
FU duration, mo, median (IQR)	30 (30-30)	30 (29-30)	0.31
Endoscopies, n, median (IQR)	6 (4-7)	3 (2-4)	0.01
Recurrence, n (%)	11 (2.8)	7 (1.4)	0.15
LGD in GEJ	3 (0.8)	1 (0.2)	0.75
Early BE neoplasia	6 (1.5)	5 (1.0)	
Advanced EAC	2 (0.5)	1 (0.2)	
Annual risk, % [95% CI]	0.11 [0.06-0.19]	0.05 [0.02-0.11]	0.15
HR recurrence* [95% CI]	1.57 [0.59-4.14]	Ref	0.37
S6.B Progression during entire FU			
Advanced neoplasia, n (%)	3 (0.7)	2 (0.4)	0.40
Annual risk, % [95% CI]	0.01 [0-0.03]	0.01 [0-0.04]	0.65
HR progression* [95% CI]	0.79 [0.11-5.84]	Ref	0.82

**Ratio for 3-monthly endoscopies versus annual endoscopies, adjusted for age, gender, length of BE, worst pathology at baseline, reflux stenosis, incident lesion lesion*

Abbreviations: BE – Barrett's esophagus; CI – confidence interval; EAC – esophageal adenocarcinoma; FU – follow-up; HR – hazard ratio; IQR – interquartile range; LGD – low-grade dyplasia; mo - months

Figure S1. Changes in outcomes over time

S1.A New patients per year

The proportion of patients treated for LGD significantly increased over time, with 14% of patients with LGD before 2013 and 36% thereafter (P 0.01; regression coefficient 2.44 [95% CI 0.82-4.07]).

S1.B Proportion of patients with ER per year

The proportion of ER for HGD patients significantly changed over time, with 47% of HGD patients undergoing ER before 2013 and 59% in the years thereafter (P0.01, regression coefficient 1.54 [95% CI 0.43-2.64]). The proportion for EAC and LGD patients did not differ significantly over time (regression coefficients , 0.01 [95% CI -0.38-0.21] for EAC and 0.28 [95% CI -1.70-2.26] for LGD).

S1B. Treatment outcomes per year

The proportion of patients with complete eradication of BE did not change over time; 5.2% of patients had successful treatment before 2013 and 6.0% thereafter (P 0.42; regression coefficient 0.01 [95%CI -0.53-0.55]).

Figure S2. Three types of recurrences during FU

Recurrences were categorized into three grades; (1) recurrent LGD in a normal appearing cardia; (2) recurrent early neoplasia with curative endoscopic treatment; (3) advanced neoplasia that exceeded boundaries for curative endoscopic re-treatment.

2.1 Recurrent LGD in GEJ

A+B) Initial C12M12 with flat HGD; C+D) CE-BE was achieved after 1 C-RFA; 2 F-RFA and touch-up APC for small remaining BE islands and biopsies from just below the cardia showed absence of IM; E) 2 years after CE-BE was established no endoscopic abnormalities were found, but biopsies from just below the cardia showed LGD. This was reproduced at the first follow-up 6 months later but not during further FU.

2.2 Recurrent early BE neoplasia

A+B) Initial BE C8M9 with a visible lesion at the 4 o'clock position that contained a well-differentiated mucosal cancer; C) after ER, 1 C-RFA and 2 F-RFA, patients achieved CE-BE and biopsies from the cardia confirmed absence of IM; D+E) 2 years after CE-BE, a small recurrent BE lesion was detected at the 6 o'clock position; and (F+G) ER was performed for a well-differentiated mucosal EAC; (H) CE-BE was re-achieved and sustained afterwards.

2.3 Progression

A) Initial C6M10 BE with a visible lesion at 3 o'clock; B) EMR was performed for mucosal, well-differentiated EAC, C) followed by circumferential RFA, 2 Focal RFA and touch-up APC for remaining small BE islands. D) CE-BE was achieved. E) 3 years after CE-BE, a recurrent BE lesion was detected at the 7 o'clock position. F+G) ER was performed for sm1-EAC with poor differentiation and positive deep resection margins. Patient was referred for esophagectomy (TxN0M0). Eight years later, recurrent EAC with lymph node metastasis had developed in the gastric tube, for which chemoradiation therapy was performed. Final outcome is pending.

Figure S3. Timing and location of recurrences

T (mo)	0	12	24	36	48	60	72	84	96	108
N	1154	977	611	455	349	317	216	148	67	33

Timing, location and size of dysplastic BE recurrences according to the initial BE length. The x-axis represents follow-up in months after the last treatment; the y-axis represents the length of the esophagus in cm with 0 being the gastro-esophageal junction. The size in the graph represents the actual size of the recurrence.

Figure S4. Timing of recurrent non-dysplastic BE tongues and BE islands

0	12	24	36	48	60	72	84	96
1154	977	611	455	349	317	216	148	67

Kaplan Meier curves for recurrent BE tongues and recurrent BE islands. Recurrent BE tongues were detected after median 38 months and BE islands after median 15 months (P 0.02). The annual risk for BE tongues was 0.4% [95% CI 0.2-0.8] in the first 2 years and 1.0% [95% CI 0.7-1.5] thereafter. BE islands had an annual risk of 3.1% [2.4-4.0] in year 1-2 and 0.8% [0.5-1.3] in the years thereafter

Figure S5. Recurrent non-dysplastic BE tongues and islands

5.1 Recurrent NDBE

A+B+C) Initial BE was C13M17 and ER was performed for a lesion containing HGD, followed by 1 Circumferential and 2 Focal RFA. D+E) CE-BE was achieved and biopsies from just below the cardia confirmed absence of intestinal metaplasia. F+G) 4 years after CE-BE was established, recurrent C0M2 BE tongue was found and biopsies showed intestinal metaplasia but no dysplasia. No progression occurred during 2 years follow-up.

5.2 Tiny islands

Several examples of tiny BE islands that were found during FU.

Figure S6. Risk for recurrence and unrelated death during long-term FU

Cumulative incidence curves for recurrence and unrelated death for the RFA durability cohort. Patients with dysplastic recurrence were censored at the moment of detection of recurrence; patients with unrelated death were censored at the date of death; all other patients were censored at the last endoscopic FU.

van Munster S, *et al. Gut* 2022; 71:265–276. doi: 10.1136/gutjnl-2020-322615
