# Supplementary figures and images for: Long-term outcomes after endoscopic treatment for Barrett’s neoplasia with radiofrequency ablation ± endoscopic resection: results from the national Dutch database in a 10-year period
Source: Gut. 2021 Mar 22;71(2):265–76. doi: 10.1136/gutjnl-2020-322615 (PMC8762001; doi:10.1136/gutjnl-2020-322615)

SFig 1A

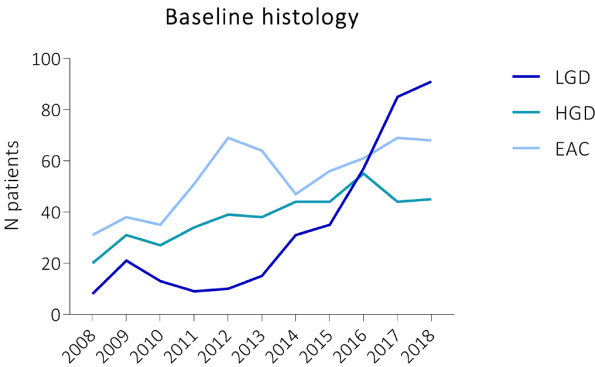

SFig 1B

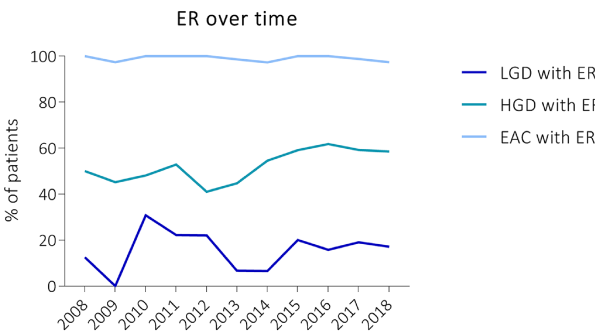

SFig 1C

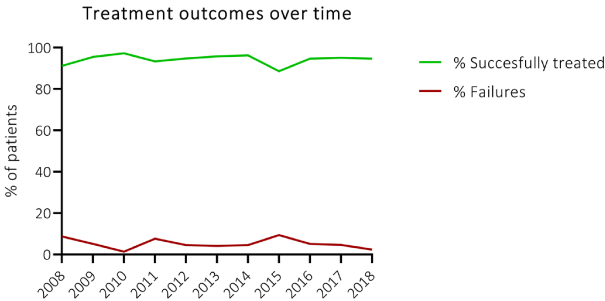

Supplement: Supplementary data [file gutjnl-2020-322615supp002.pdf]

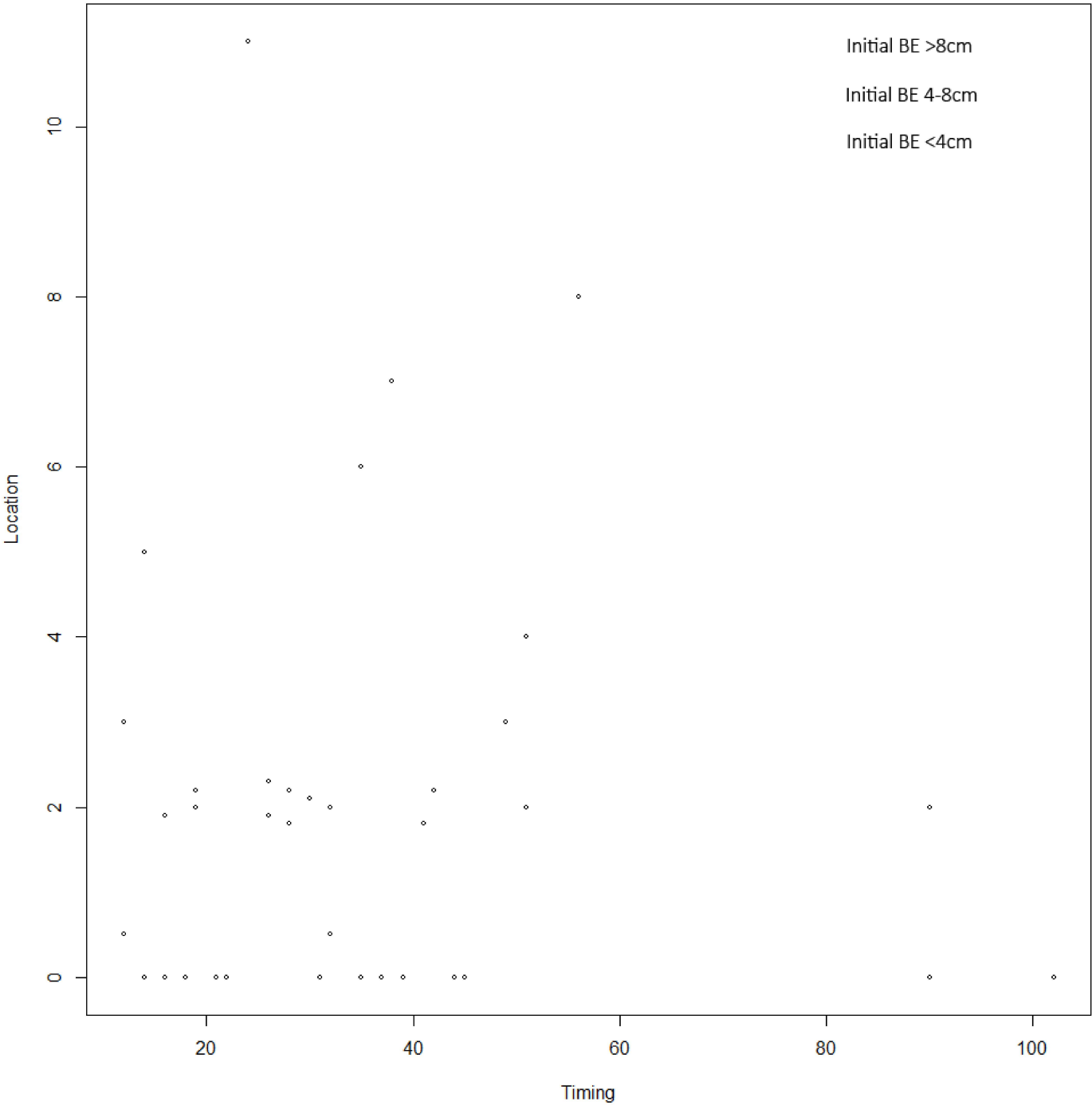

Supplement: Supplementary data [file gutjnl-2020-322615supp004.pdf]

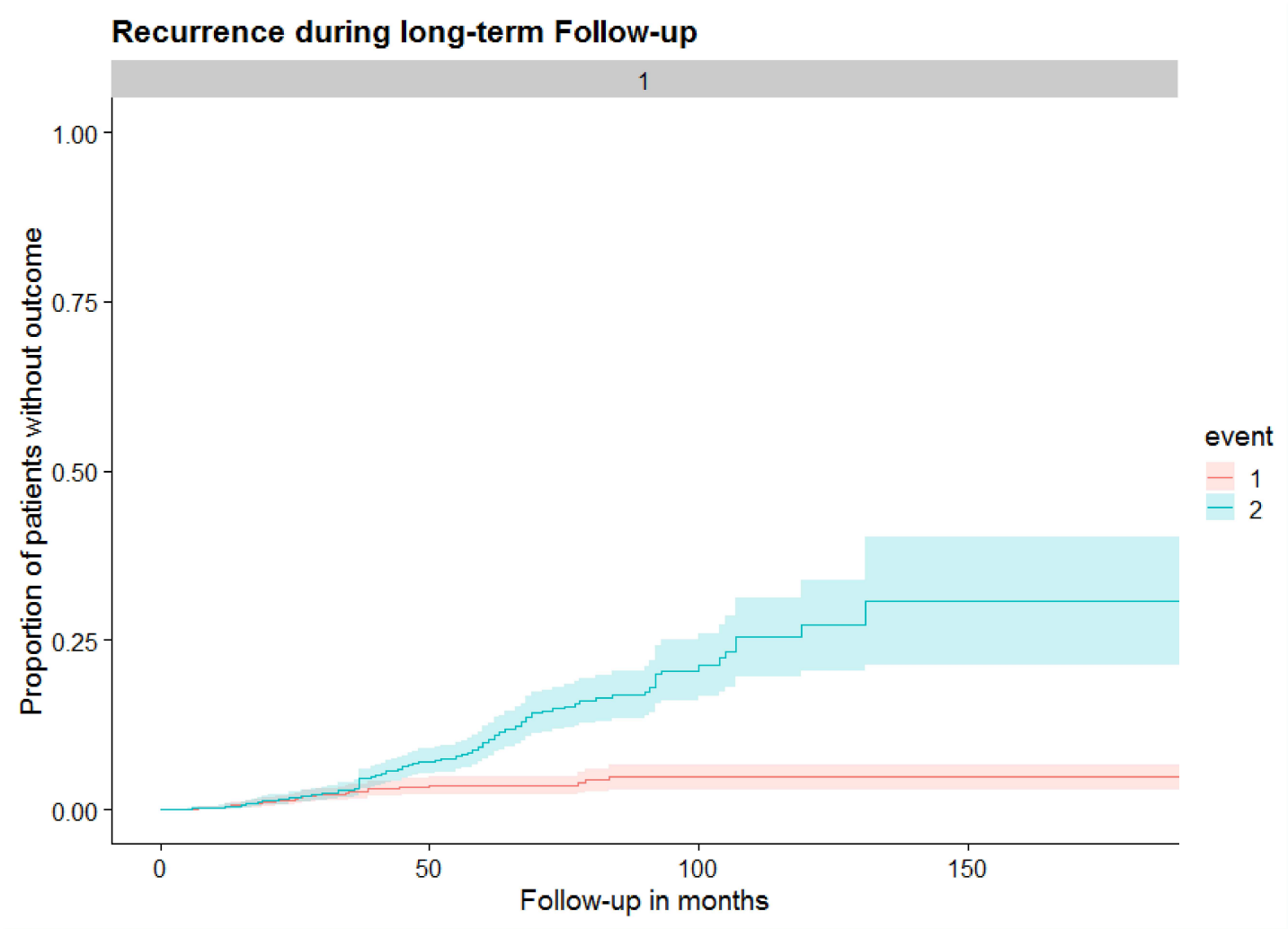

Supplement: Supplementary data [file gutjnl-2020-322615supp007.pdf]
