# Supplementary material for: Long-term outcomes after endoscopic treatment for Barrett’s neoplasia with radiofrequency ablation ± endoscopic resection: results from the national Dutch database in a 10-year period
Source: Gut. 2021 Mar 22;71(2):265–76. doi: 10.1136/gutjnl-2020-322615 (PMC8762001; doi:10.1136/gutjnl-2020-322615)
Supplement: Supplementary data [file gutjnl-2020-322615supp005.pdf]

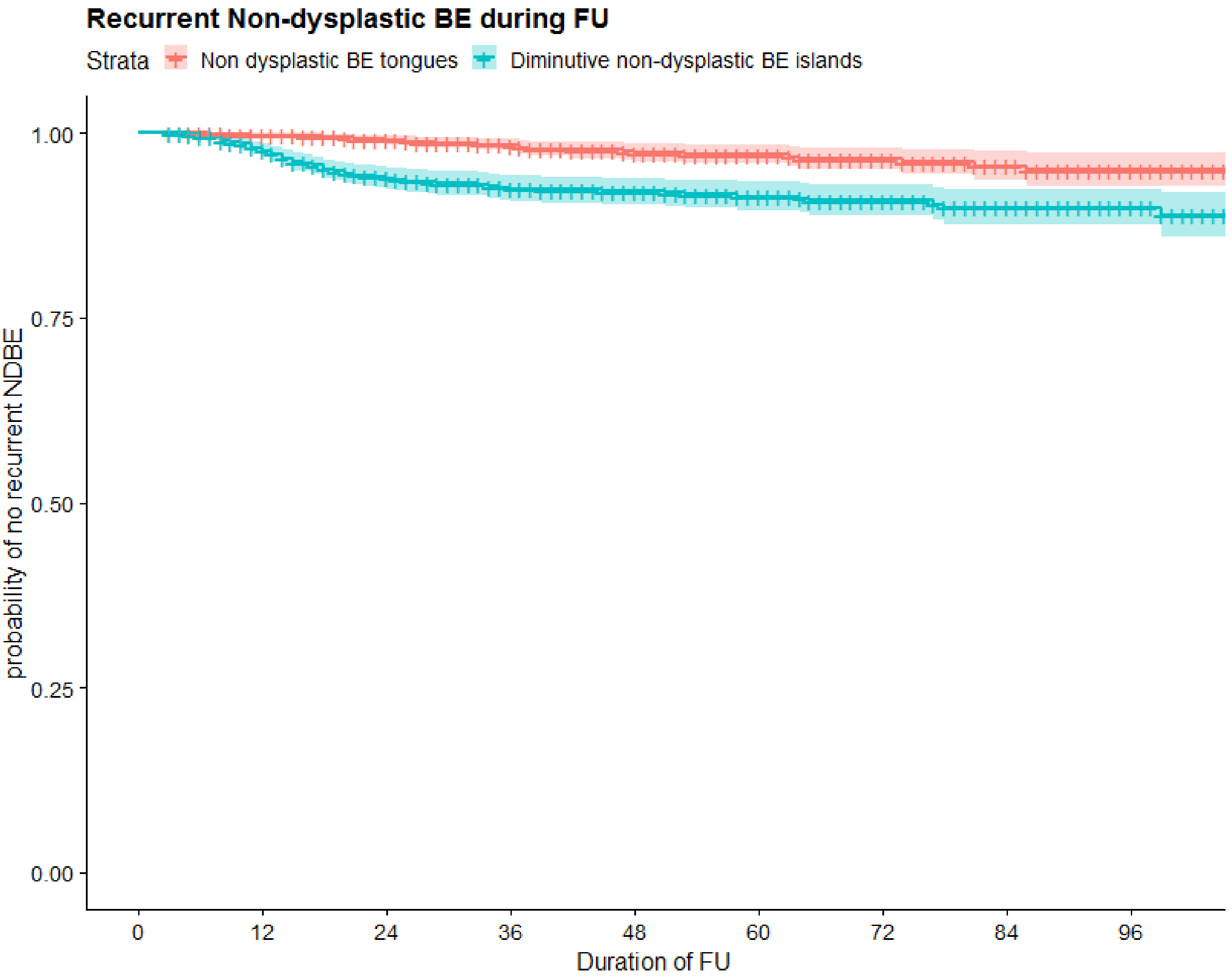
Recurrent Non-dysplastic BE during FU
Strata
Non dysplastic BE tongues
Diminutive non-dysplastic BE islands
probability of no recurrent NDBE
1.00
0.75
0.50
0.25
0.00
0
12
24
36
48
60
72
84
96
Duration of FU
